# PolyICLC Exerts Pro- and Anti-HIV Effects on the DC-T Cell Milieu *In Vitro* and *In Vivo*

**DOI:** 10.1371/journal.pone.0161730

**Published:** 2016-09-07

**Authors:** Meropi Aravantinou, Ines Frank, Magnus Hallor, Rachel Singer, Hugo Tharinger, Jessica Kenney, Agegnehu Gettie, Brooke Grasperge, James Blanchard, Andres Salazar, Michael Piatak, Jeffrey D. Lifson, Melissa Robbiani, Nina Derby

**Affiliations:** 1 Center for Biomedical Research, Population Council, New York, NY, United States of America; 2 Linköping University, Linköping, Sweden; 3 Aaron Diamond AIDS Research Center, Rockefeller University, New York, NY, United States of America; 4 Tulane National Primate Research Center, Tulane University Health Sciences Center, Covington, LA, United States of America; 5 Oncovir, Inc., Washington, DC, United States of America; 6 AIDS and Cancer Virus Program, Leidos Biomedical Research, Inc., Frederick National Laboratory, Frederick, MD, United States of America; Harvard Medical School, UNITED STATES

## Abstract

Myeloid dendritic cells (mDCs) contribute to both HIV pathogenesis and elicitation of antiviral immunity. Understanding how mDC responses to stimuli shape HIV infection outcomes will inform HIV prevention and treatment strategies. The long double-stranded RNA (dsRNA) viral mimic, polyinosinic polycytidylic acid (polyIC, PIC) potently stimulates DCs to focus Th1 responses, triggers direct antiviral activity *in vitro*, and boosts anti-HIV responses *in vivo*. Stabilized polyICLC (PICLC) is being developed for vaccine adjuvant applications in humans, making it critical to understand how mDC sensing of PICLC influences HIV infection. Using the monocyte-derived DC (moDC) model, we sought to describe how PICLC (vs. other dsRNAs) impacts HIV infection within DCs and DC-T cell mixtures. We extended this work to *in vivo* macaque rectal transmission studies by administering PICLC with or before rectal SIVmac239 (SIVwt) or SIVmac239ΔNef (SIVΔNef) challenge. Like PIC, PICLC activated DCs and T cells, increased expression of α_4_β_7_ and CD169, and induced type I IFN responses *in vitro*. The type of dsRNA and timing of dsRNA exposure differentially impacted *in vitro* DC-driven HIV infection. Rectal PICLC treatment similarly induced DC and T cell activation and pro- and anti-HIV factors locally and systemically. Importantly, this did not enhance SIV transmission *in vivo*. Instead, SIV acquisition was marginally reduced after a single high dose challenge. Interestingly, in the PICLC-treated, SIVΔNef-infected animals, SIVΔNef viremia was higher, in line with the importance of DC and T cell activation in SIVΔNef replication. In the right combination anti-HIV strategy, PICLC has the potential to limit HIV infection and boost HIV immunity.

## Introduction

Myeloid dendritic cells (mDCs) orchestrate immune responses to infections at mucosal sites. Immature mDCs sample mucosal surfaces for invading pathogens and upon encounter, decrease their sentinel function in favor of T cell interaction and activation to initiate and regulate effective immunity. mDCs are among the first leukocytes to encounter HIV during sexual transmission [1] and are crucial in establishing antiviral immunity against HIV [2, 3]. Yet, HIV has co-opted the sentinel and immunoregulatory functions of mDCs to disseminate virus and expand infection [3–7]. Immature mDCs isolated from blood [8], immature monocyte-derived DCs (moDCs) that are used to model mDCs *in vitro* [9–12], and Langerhans cells (LCs) [13] can all capture HIV. They efficiently transfer infectious particles to CD4^+^ T cells across the DC-T cell infectious synapse in *trans* while immature moDCs (iDCs) also become productively infected at a low level, supplying virus to T cells in *cis* [2–6, 8–12]. *Cis* transfer is thought to contribute especially to long-term viral transmission [11, 12, 14, 15].

mDC responses to stimuli differentially shape innate and adaptive immunity and influence HIV susceptibility [2, 6, 11, 16]. Diverse microbial products, cytokines, endogenous ligands, and pathogens mature mDCs to differing degrees and with different qualities, giving rise to diverse DC phenotypes that variably direct T cell fate, HIV capture, and the outcome of HIV infection in DCs and the CD4^+^ T cells they encounter [2, 11, 13, 17–27]. Another layer of complexity in the outcome is imparted by the timing of DC maturation with respect to HIV and T cell exposure [17, 28].

Polyinosinic polycytidylic acid (polyIC, shortened throughout to PIC) is a valuable tool for dissecting the nuances of DC-driven HIV transmission and replication and a potent immunostimulatory agent for focusing Th1 responses *in vivo* [29–31]. We have previously shown that this long dsRNA viral mimic completely shuts down HIV infection of virus-bearing iDCs [32] through a mechanism involving type I IFN-induced activation of APOBEC3G (A3G) and A3A [32–35]. However, PIC-matured DCs (picDCs) and picLCs capture more HIV than their immature counterparts and more efficiently drive infection in T cells in *trans* [13, 16]. picDCs were recently shown to express increased levels of the interferon (IFN)-inducible macrophage marker CD169, and this facilitated HIV capture [18, 19, 36]. DCs matured with lipopolysaccharide (LPS) also captured HIV in a CD169-dependent manner, resulting in increased *trans* infection of autologous CD4^+^ T cells and T cell lines [18, 19]. Though a similar mechanism has been surmised for both TRIF-dependent TLR ligands [19], the importance of CD169-mediated HIV capture in picDC-driven HIV infection was not reported [13, 19].

Despite an expansive body of research, PIC is not suitable for clinical development as it is subject to serum nuclease activity in primates *in vivo* [37]. PolyICLC (PICLC) is a clinical grade modified formulation of PIC (stabilized with poly-L-lysine and carboxymethylcellulose [38]) that preserves immunomodulatory activities [37, 39, 40]. It induces mucosal and systemic innate antiviral responses in rhesus macaques [41, 42] and humans [43], has demonstrated safety and anti-neoplastic and IFN-inducing activity in humans, and is actively being developed as an adjuvant for antiviral and anti-cancer vaccines and therapeutics [29, 30, 37, 43–46] as well as a potential HIV latency reversing agent [47]. In macaques, PICLC induces type I IFN [38], possesses antiviral activity [48] and has been dosed as an adjuvant [25, 27, 29, 30, 41, 42, 49, 50]. However, whether or not prophylactic use of PICLC can affect SIV transmission directly *in vivo* has not been examined.

Depending on their length and structure, dsRNAs can bind multiple pattern recognition receptors (PRRs; e.g. TLR3, MDA-5, and RIG-I). PIC and PICLC are both recognized ligands for TLR3 and MDA-5 [37, 51–54] though this is less extensively characterized for PICLC [55], and another PIC derivative developed for clinical use, polyIC_12_U, only binds TLR3 [37, 50, 55, 56]. It is possible that PICLC may stimulate mature DCs with different characteristics from the parent PIC [37, 53, 56] and may promote divergent outcomes for HIV replication. Another dsRNA, polyadenylic polyuridylic acid (polyAU, PAU), similarly promotes DC and T cell activation, directs Th1-focused antigen-specific immune responses in mice, and possesses anti-tumor activity in humans [57, 58]. However, like polyIC_12_U, PAU signals only through TLR3 and additionally has not been studied in the context of DC-driven HIV transmission. The effects of PICLC vs. other dsRNAs on the DC-T cell environment need to be characterized *in vitro* to best understand the biology pertinent to clinical progression of PICLC.

Herein, we sought to characterize how PICLC (vs. other dsRNAs) matures DCs and impacts viral capture and infection therein and in the DC-T cell milieu *in vitro*. In order to assess the importance of DC function in mucosal HIV acquisition *in vivo* and potentially identify a role for PICLC-mediated mDC maturation in tipping the balance between protection and transmission, we examined how PICLC impacts rectal SIV transmission in macaques, a model which recapitulates the role of mDCs in HIV infection [5]. Since Nef facilitates HIV replication in the DC-T cell milieu [59, 60], and SIVΔNef requires mature DCs for replication in the DC-resting T cell milieu and T cell activation in iDC-T cell mixtures [61], we compared the effects of PICLC on SIV containing wild type Nef (SIVwt) with these effects on an attenuated virus lacking full length Nef, SIVΔNef. The results reveal complex differential effects of PICLC *in vitro* (viral inhibitory vs. enhancing) that depended on when PICLC was added to DC-T cell co-cultures. *In vivo* findings largely corroborated these *in vitro* results, suggested potentially diverging effects of PICLC on SIVwt and SIVΔNef replication, and highlighted the importance of CD169 as a biomarker in HIV pathogenesis although not as a predictor of mucosal acquisition of infection.

## Materials and Methods

### Viruses

HIV_Bal_ (HIV, lots P4143, P4237, and P4239) stocks were provided by the Biological Products Core of the AIDS and Cancer Virus Program, Frederick National Laboratory, Frederick, MD). Stocks were sucrose density gradient purified [62] and stored at -80°C, and titers were confirmed by titration on TZM.bl cells (ATCC, Mannassas, VA) [63].

Stocks of SIVwt and SIVΔNef were grown for these studies in freshly isolated rhesus macaque peripheral blood mononuclear cells (PBMCs, obtained from SIV-uninfected macaques assigned to these studies and housed at Tulane National Primate Research Center (TNPRC)–see below) from single donors [23, 64]. The cells (10^6^ cells/ml) were cultured in R10 media (RPMI 1640 (Cellgro, Fisher Scientific, Springfield, NJ) containing 10% fetal bovine serum (FBS, Gibco, Life Technologies, Waltham, MA) and 100 U/ml penicillin/ 100 μg/ml streptomycin (Gibco) supplemented with 5 μg/ml phytohaemagglutinin (PHA, Sigma-Aldrich, St. Louis, MO) for 3 days at 37°C, washed, and cultured an additional 3 days in R10 with 10% IL-2 (Schiapparelli Biosystems, Fairfield, NJ). Cells were adjusted to 10^6^ cells/ml and inoculated with 610 50% tissue culture infectious doses (TCID_50_) stock SIV/10^6^ cells for SIVwt and 1220 TCID_50_ stock SIV/10^6^ cells for SIVΔNef. Both stocks were used in prior studies [20, 61] and re-titered prior to inoculating cells for new stocks. Cell counts were adjusted to 10^6^ cells/ml on day 4 and day 7 post-infection, and the whole supernatant containing virus was harvested on day 8 and centrifuged at 1500 rpm for 10 minutes to remove cellular debris. Aliquots (1ml) were stored at -80°C. Virus titer was determined in CEMx174 (ATCC) cells by p27 ELISA quantification (ZeptoMetrix, Buffalo, NY) and syncytia scoring after 14 days with the calculation method of Reed and Meunch [65].

### dsRNAs

The dsRNAs utilized for these studies were PIC (InvivoGen, San Diego, CA), PICLC (Oncovir, Washington, DC), and PAU (InvivoGen). Their sizes (alongside the size of low molecular weight (LMW) PIC (InvivoGen)) were characterized by electrophoresis on 0.8% agarose gels in comparison with the 1 kb Plus DNA ladder (Invitrogen, Life Technologies, Waltham, MA).

### Cells for *in vitro* experiments

The CD14^+^ fraction of PBMCs was isolated from buffy coats of anonymous healthy human blood donors (New York Blood Center, New York, NY) using the MACS system (Miltenyi, San Diego, CA) as previously described [22, 32]. iDCs were generated from these CD14^+^ cells as described [22, 32]. After 5 days of culture in R1 media (RPMI 1640 containing 2 mM L-glutamine (Gibco), 10 mM HEPES (Gibco), 50 μM 2-mercaptoethanol (Sigma), 100 U/ml penicillin/100 μg/ml streptomycin, and 1% heparinized human plasma (Innovative Research, Novi, MI)) supplemented with recombinant human interleukin-4 (IL-4, 100 U/ml; Biosource, Atlanta, GA) and recombinant human granulocyte-macrophage colony-stimulating factor (GM-CSF, 1000 U/ml; Biosource), iDCs were cultured a further 48 hours in R1 with GM-CSF/IL-4 while mature moDCs were generated by continuing the culture for 48 hours in R1 containing stimuli: 10 μg/ml PIC to generate picDCs, 10 μg/ml PICLC to generate piclcDCs, and in some donors, 10 μg/ml PAU to generate pauDCs. Immature and mature moDCs were collected and their phenotype and purity analyzed by flow cytometry on a BD LSRII (BD Biosciences, San Jose, CA) using software from Diva (BD) and FlowJo (Ashland, OR) for acquisition and analysis, respectively. moDCs routinely contained less than 2% contaminating CD3^+^ cells.

Autologous CD14^-^ cells from the buffy coat PBMCs (NY Blood Center) were cultured for 6 days in R1 supplemented with 10 U/ml of IL-2 (Preclinical Repository, National Cancer Institute at Frederick, NCI-Frederick, MD) at 20 x 10^6^ cells/ml before CD4^+^ T cells were isolated by negative selection using the human CD4^+^ T cell isolation MACS system (Miltenyi). T cell phenotype and purity were analyzed by flow cytometry on the LSRII. Freshly isolated CD4^+^ T cells were cultured overnight in R1 supplemented with 10 U/ml of IL-2 at 20 x 10^6^ cells/ml.

When human or macaque PBMCs (human PBMCs from buffy coats of human donors, NY Blood Center; macaque PBMCs from blood of macaques housed at TNPRC for these studies) were directly subjected to dsRNA stimulation, the isolated, washed PBMCs were re-suspended at 2 x 10^6^ cells/ml and plated in 96-well flat-bottom tissue culture plates in 0.2 ml R1 containing 10 μg/ml PIC or PICLC vs. media alone. After 24 hours, cells from replicate wells were pooled and processed for flow cytometry or reverse transcriptase quantitative PCR (RT-qPCR).

### DC-T cell assays

DCs (iDCs, picDCs, piclcDCs, pauDCs) derived as described above were pulsed by incubating with HIV (8 x 10^4^ TCID_50_/10^6^ DCs) for 1.5 hours at 37°C and washing as previously described [32]. In some experiments, freshly pulsed cells were processed for flow cytometry. Alternatively, pulsed DCs were re-plated in 96-well flat-bottom plates alone (3 x 10^5^ cells/0.2 ml well volume) or with autologous CD4^+^ T cells (1 x 10^5^ DCs and 3 x 10^5^ T cells). Cultures containing mature DCs were in R1 while iDC and iDC-T cell co-cultures had GM-CSF and IL-4 added every 2 days. IL-2 (10 U/ml) was added every 2 days to all DC-T cell co-cultures. To evaluate the effect of stimuli on iDC-containing cultures, PIC, PICLC, or PAU (10 μg/ml) were added once immediately upon plating (no GM-CSF/IL-4). After 7 days, HIV infection was measured by DNA quantitative PCR (qPCR) on lysed cells (HIV *gag* vs. *ALB* (albumin) as a cell number control) [32]. Within each experiment, cells from each donor under each condition were cultured in 2–4 replicates, and one independent PCR reaction was run on each replicate to derive a mean for that donor. The mean of replicates for each donor is plotted in the figures. In some cases, co-cultures were harvested after 24 hours and processed for flow cytometry or RT-qPCR.

To infect human DCs, T cells, and DC-T cell mixtures with HIV in the absence of pulsing, cells, virus, and stimuli were added together in 96-well flat-bottom plates at the final concentrations described above without washing. When CD4^+^ T cells were infected in the absence of DCs, 50 U/ml IL-2 was added to the cultures every 2 days. HIV infection was measured by qPCR after 7 days as above.

### Ethics statement

Adult male Indian rhesus macaques (*Macaca mulatta*; mean age: 6.8 years, range: 4.4–9.4 years; mean weight: 10.6 kg, range: 6.6–13.8 kg) that tested negative by serology and virus-specific PCR for SIV, SRV, Herpes B, and STLV-1 were selected for these studies. Animal care complied with the regulations stated in the Animal Welfare Act [66] and the Guide for the Care and Use of Laboratory Animals [67], at Tulane National Primate Research Center (TNPRC, Covington, LA). All macaque studies were approved by the Institutional Animal Care and Use committee (IACUC) of TNPRC for macaques (OLAW Assurance #A4499-01) and complied with TNPRC animal care procedures. TNPRC receives full accreditation by the Association for Accreditation of Laboratory Animal Care (AAALAC #000594). Animals were socially housed indoors in climate-controlled conditions and were monitored twice daily by a team of veterinarians and technicians to ensure their welfare. Any abnormalities, including changes in appetite, stool, and behavior, were recorded and reported to a veterinarian. They were fed commercially prepared monkey chow twice daily. Supplemental foods were provided in the form of fruit, vegetables, and foraging treats as part of the TNPRC environmental enrichment program. Water was available continuously through an automated watering system.

Veterinarians at the TNPRC Division of Veterinary Medicine have established procedures to minimize pain and distress through several means in accordance with the recommendations of the Weatherall Report. Prior to all procedures, including blood draws, macaques were anesthetized with ketamine-HCl (10 mg/kg) or tiletamine/zolazepam (6 mg/kg). Preemptive and post-procedural analgesia (buprenorphine 0.01 mg/kg) was administered for procedures that could cause more than momentary pain or distress in humans undergoing the same procedures. Macaques were euthanized in this study only if and when they became sick (TNPRC IACUC-approved humane endpoint criteria) using methods consistent with recommendations of the American Veterinary Medical Association (AVMA) Panel on Euthanasia and per the recommendations of the IACUC. For euthanasia, animals were anesthetized with tiletamine/zolazepam (8 mg/kg) and given buprenorphine (0.01 mg/kg) followed by an overdose of pentobarbital sodium. Death was confirmed by auscultation of the heart and pupillary dilation. All animals that remained healthy at the conclusion of the study were reassigned to other studies.

### Animal treatments and specimen collection

#### PICLC acute effects

Eleven SIV-uninfected macaques were used to evaluate acute immune changes following rectal PICLC application. To set the baseline, the macaques were atraumatically dosed rectally with 1 ml PBS (Gibco) twice, 24 hours apart. Four hours after the second dose, all 11 macaques were bled and from 6 animals, rectal biopsies were collected. Twenty-four hours after PBS application, all 11 macaques were bled again, and the 5 animals not mucosally sampled at 4 hours had rectal biopsies collected. To acquire data replicates, another PBS application followed by 4 and 24 hour sampling was performed in the same way after mucosal healing (9 weeks following the first set of biopsies). After healing of the second biopsy (4 weeks post-biopsy), all 11 macaques received rectally 2 doses of 1 mg PICLC 24 hours apart. Each dose consisted of 1 ml of 1 mg/ml PICLC prepared in PBS. Post-PICLC blood and biopsies were collected 4 and 24 hours after the second dose in the same way as after PBS treatments. A second PICLC application and sampling was performed 4 weeks after the first. In a separate set of animals, PICLC was administered using 2 different regimens: single doses of 2 mg or 4 mg. Baseline pre-treatment rectal swabs and biopsies were collected 5 weeks before treatment, with sampling again 24 hours after treatment (pre vs. post). Samples were available for study from 4 of the “2 mg” and 2 of the “4 mg” macaques.

All blood and biopsies were collected and shipped to the Population Council in New York overnight and processed immediately on arrival as previously described [42, 64]. Plasmas were isolated and stored at -80°C [64]. Isolated PBMCs [23, 64] were used immediately for flow cytometry. Rectal swabs were cleared by centrifugation and stored at -80°C [42]. Rectal biopsies (1.5mm x 1.5mm) were transported in L-15 media (HyClone Laboratories, Inc., Logan, UT) supplemented with 10% FBS and 100 U/ml penicillin/100 μg/ml streptomycin and washed. Half of the tissue pieces from the first 11 animals were placed in RNALater (Qiagen, Limburg, Netherlands) overnight at 4°C before being transferred to storage at -20°C. The remaining pieces were digested with collagenase II (0.5 mg/ml; Invitrogen), hyaluronidase (1 mg/ml; Sigma), and DNase (1 mg/ml; Roche, Basel, Switzerland) in R10 for up to 2 hours shaking at 37°C. Released cells were washed, passed through a 40 μm cell strainer, and re-suspended in FACS buffer (PBS supplemented with 5% FBS and 0.1% sodium azide, pH 7.2–7.4) for flow cytometry. For these 11 animals, rectal lymphocytes were enriched by centrifugation through Percoll (40% Percoll [Sigma], 60% FBS in PBS) for 20 minutes at room temperature before additional washing and passage through the cell strainer. Rectal biopsies from the latter 6 “pre vs. post” animals were transported in L-15, digested as above (without Percoll), washed, and stored as a dry pellet of 5–10 x 10^6^ cells/tube at -80°C.

#### SIVwt challenge study

Twenty-two SIV-naïve macaques were used to test potential *in vivo* antiviral effects (acquisition and replication) of PICLC against SIVwt challenge (Table 1). PICLC (1 ml of 1 mg/ml in PBS as in acute effects study) was atraumatically administered rectally twice 24 hours apart to 14 of the 22 macaques. Seven of these 14 were rectally challenged with 3000 TCID_50_ SIVmac239 at the same time as the second dose (virus and PICLC mixed together in 1 ml total volume, “coincident”). The other 7 were rectally challenged 24 hours after the second dose with 3000 TCID_50_ in 1 ml (“24h pre”). Of the 8 control macaques, 4 received 1 ml PBS twice 24 hours apart and were challenged 24 hours after the second dose (PBS), and the other 4 received no treatment before challenge. The animals were followed for 20–24 weeks within this study except for one, FF86, which had to be euthanized at week 18 due to simian AIDS (endpoint criterion for euthanasia). Survival time for all SIV-infected macaques is shown in Table 1. Blood and rectal biopsies were collected periodically throughout and processed as above (no Percoll). Overnight-shipped rectal biopsies were either digested immediately to obtain cells for flow cytometry or washed and placed in RNALater overnight at 4°C before being transferred to -20°C for storage. Viral loads were determined in plasma from the animals by RT-qPCR as previously described [68, 69]. Infection was defined as two consecutive time points with plasma viremia >100 copies/ml or any viremia >1000 copies/ml, consistent with previously defined criteria [70, 71].

**Table 1:** Rhesus macaques used in challenge studies.

Animal ID	Treatment	Virus	SIV Δnef infection^a^	SIVwt infection	CD4 Count			Survival time of SIV^+^ (weeks post-infection)^c^
					BL	Wk 3–4^b^	Wk 24	
FI36	PICLC coincident	SIVwt	ND^d^	+	807	496	518	35
FH36	PICLC coincident	SIVwt	ND	-	801	1043	907	na^e^
DG72	PICLC coincident	SIVwt	ND	+	804	334	192	36
FE87	PICLC coincident	SIVwt	ND	-	850	1280	869	na
FH30	PICLC coincident	SIVwt	ND	+	1215	596	278	46
EK35	PICLC coincident	SIVwt	ND	+	1556	1123	966	34
CP50	PICLC coincident	SIVwt	ND	-	492	575	541	na
EJ97	PICLC 24h pre	SIVwt	ND	+	1163	824	681	130
FH32	PICLC 24h pre	SIVwt	ND	-	630	1019	752	na
EB34	PICLC 24h pre	SIVwt	ND	-	747	960	580	na
FF57	PICLC 24h pre	SIVwt	ND	+	1251	626	538	43
FE39	PICLC 24h pre	SIVwt	ND	-	850	785	482	na
FB86	PICLC 24h pre	SIVwt	ND	+	846	550	325	114
FT04	PICLC 24h pre	SIVwt	ND	+	715	842	482	32
FF75	None	SIVwt	ND	+	525	543	NA^f^	24
FD64	None	SIVwt	ND	+	769	410	99	37
FC25	None	SIVwt	ND	+	1387	1001	272	59
FN88	None	SIVwt	ND	+	416	882	347	33
EN40	PBS	SIVwt	ND	-	853	860	1063	na
CP74	PBS	SIVwt	ND	-	425	329	451	na
FF86	PBS	SIVwt	ND	+	567	527	NA	18
CM68	PBS	SIVwt	ND	+	460	259	223	114
CR30	PICLC 24h pre	SIV ΔNef^g^	-	+	223	234	97	42
GA73	PICLC 24h pre	SIV ΔNef	-	+	967	1076	387	76
GH44	PICLC 24h pre	SIV ΔNef	+	-	1023	1022	808	112
GI09	PICLC 24h pre	SIV ΔNef	+	-	685	689	541	112
GI67	PICLC 24h pre	SIV ΔNef	+	-	1748	1260	782	112
GK52	PICLC 24h pre	SIV ΔNef	-	+	812	681	312	82
GK53	PICLC 24h pre	SIV ΔNef	-	-	NA	1138	805	na
FE67	None	SIV ΔNef	+	-	1426	934	1011	177
FF90	None	SIV ΔNef	-	-	709	841	791	na
FG74	None	SIV ΔNef	+	+	773	806	187	29
FP31	None	SIV ΔNef	+	-	716	369	644	177
GM84	PBS	SIV ΔNef	+	-	1016	1224	1306	112
GN96	PBS	SIV ΔNef	-	-	443	531	251	na
GP34	PBS	SIV ΔNef	+	+	847	605	522	82

^a^For each virus, positive infection status determined by two consecutive positive time points above 100 copies/ml or any time point above 1000 copies/ml [70, 71].

^b^SIVwt-challenged macaques had CD4 count at Wk 3. SIVΔNef-challenged macaques had CD4 count at Wk 4.

^c^Macaques were monitored closely within this study for 20–24 weeks and then SIVwt-infected animals continued to be cared for until they were euthanized due to simian AIDS. Uninfected animals were transferred to other studies. SIVΔNef-infected macaques were transferred to another study in which they were necropsied on a set date: GH44, GI09, GI67, GM84.

^d^ND indicates no SIVΔNef challenge was performed.

^e^na indicates not applicable.

^f^NA indicates the sample was not available.

^g^All macaques challenged with SIVΔNef were subsequently challenged with SIVwt 12 weeks later.

#### SIVΔNef challenge study

Fourteen SIV-naïve macaques were used to test the antiviral effects (acquisition and replication) of PICLC against SIVΔNef challenge (Table 1). As in the “24h pre” group within the SIVwt study, PICLC (7 macaques) vs. PBS (7 macaques) was administered twice 24 hours apart before all animals were challenged rectally with 3000 TCID_50_ SIVmac239ΔNef 24 hours after the second dose. To also explore whether PICLC impacted SIVΔNef-induced immune responses that protect from subsequent exposure to SIVwt, we challenged the SIVΔNef-infected and uninfected macaques rectally with 3000 TCID_50_ SIVwt 12 weeks after SIVΔNef challenge. Challenging SIVΔNef-uninfected macaques with SIVwt alongside provided an internal control for SIVwt infection. Samples were collected and the animals were followed for 20–24 weeks post-SIVwt as described above. Plasma viral loads were determined by discriminatory RT-qPCR in nef [24, 72]. Survival time is noted in Table 1. None of these 14 animals became sick during the study follow up period.

### Flow cytometry

Cell suspensions (human: DCs, DC-T cell mixtures, CD4^+^ T cells, PBMCs; macaque: PBMCs, rectal cells) were stained with the LIVE/DEAD Aqua viability dye (Aqua; Molecular Probes, Life Technologies, Carlsbad, CA) according to the manufacturer’s instructions. DCs and DC-T cell mixtures were blocked with 1 μg/sample human IgG (Jackson ImmunoResearch, West Grove, PA) before staining when using a DC panel. Surface staining was performed for 20 minutes at 4°C after which cells were washed and fixed in 2% paraformaldehyde. When anti-CCR5 was included in the panel, the cells were incubated with the antibody mix for 5 minutes at room temperature before being transferred to 4°C. Antibodies (listed below) were all from BD Biosciences unless noted.

Human DCs were surface stained with combinations of: anti-HLA-DR Qdot™605 (Invitrogen) or BV605, anti-CD25 PE-Cy7, anti-CD80 APC-Alexa780 or APC-H7, anti-CD83 PE (Beckman Coulter, Brea, CA), anti-CD86 PE or eFluor710 (eBioscience, San Diego, CA), anti-CD206 PE, anti-CD209 APC, anti-CD11c PE-Cy7 or AF700 (eBioscience), anti-CD4 PerCP-Cy5.5, anti-CCR5 PE-Cy7 (antibody from NIH AIDS Reference and Reagent Program conjugated to PE-Cy7 in house with a kit from Innova Biosciences [Cambridge, United Kingdom] according to the manufacturer’s instructions), anti-α_4_β_7_ PE or APC (Non-human Primate Reagent Program), anti-CD103 FITC (eBioscience), anti-MAdCAM-1 PE (BioRad, Philadelphia, PA), and anti-CD169 PE or APC (clones 7D2 and 7–239 from Santa Cruz Biotechnologies, Dallas, TX and Biolegend, San Diego, CA, respectively). Anti-CD3 V450 was used to measure T cell contamination of DC preparations.

Human CD4^+^ T cells were surface stained with combinations of: anti-CD3 V450, anti-CD4 PerCP-Cy5.5, anti-CD25 APC, anti-HLA-DR Qdot™605 or BV605, anti-CD45RO APC, anti-CCR5 PE-Cy7 (prepared as for DC staining), anti-α_4_β_7_ PE, and anti-CD69 APC-Cy7. Anti-CD8 APC-Cy7 was used to measure CD8^+^ T cell contamination of isolated CD4^+^ T cells.

Intracellular staining to detect HIV p24 was performed following surface staining. HIV-pulsed DCs (immediately after the pulse or after an overnight incubation) or DC-T cell mixtures (after overnight incubation) were surface stained as above, and then cell membranes were fixed and permeabilized by 20 minutes incubation at 4°C with Fix/Perm buffer (BD), and cells were incubated with anti-HIV-1 p24 PE or FITC (KC57, Beckman Coulter) in PermWash buffer (BD) for 20 minutes at room temperature. Cells were washed, re-suspended in PermWash buffer, and flow cytometry data were acquired the same day. For p24 detection, non-pulsed cells labeled with anti-p24 were used as the control instead of pulsed cells labeled with isotype [10].

Macaque blood DCs were examined by surface staining PBMCs with Aqua followed by the lineage-excluding combination of anti-CD3, anti-CD14, and anti-CD20 all in FITC (Lin), anti-HLA-DR PerCP-Cy5.5, anti-CD11c PE-Cy7, anti-CD123 PE or PerCP-Cy5.5, anti-CD80 APC-H7, and anti-CCR7 APC. Macaque blood and rectal T cells were examined by surface staining PBMC or rectal cell suspensions with Aqua followed by anti-CD3 V450, anti-CD4 PerCP-Cy5.5, anti-CD69 APC-Cy7, anti-CCR7 APC, CD95-FITC, and anti-α_4_β_7_ PE.

The gating strategy for human DCs was large cells (FSC-A/SSC-A) → singlets (FSC-A/FSC-H) → live cells (Aqua^-^) → HLA-DR^+^. The gating strategy for CD4^+^ T cells was small cells (FSC-A/SSC-A) → singlets → live cells → CD4^+^ T cells (CD3^+^CD4^+^). For DC-T cell co-cultures, CD4^+^ T cells were also examined in conjugates by gating on large cells (FSC-A/SSC-A) → live cells → CD4^+^ T cells (CD3^+^CD4^+^). CD8^+^ T cells (from macaque *in vivo* samples only) were gated as CD3^+^CD4^-^ live cells. The gating strategy for human and macaque blood mDCs was lymphocytes (FSC-A/SSC-A) → singlets → live cells → DCs (CD3^-^CD14^-^CD20^-^ [Lin^-^] HLA-DR^+^) → mDCs (CD11c^+/hi^CD123^-^). Blood pDCs (macaque) were gated as CD11c^-^CD123^+^ DCs. For all *in vitro* experiments, at least 100,000 and up to 200,000 live cells (DCs) or CD4^+^ T cells (DC-T cell co-cultures) were acquired. From *in vivo* samples, 50,000 Lin^-^HLA-DR^+^ DCs or 100,000 CD4^+^ T cells could usually be acquired.

### RT-qPCR

RNA was isolated from human DC, DC-T cell, and PBMC dry pellets using the RNeasy mini kit (Qiagen) and from macaque rectal biopsy specimens stored in RNALater using the RNeasy tissue kit (Qiagen) according to the manufacturer’s instructions. Qiashredder columns (Qiagen) were used to disrupt DCs, DC-T cell mixtures, and PBMCs. Rectal tissues were thawed, washed, and homogenized using a FastPrep bead mill homogenizer with lysing matrix D (MP Biomedicals, Irvine, CA) as previously described [42] prior to RNA isolation. Total RNA was subjected to on-column DNA digestion with RNase-free DNase (Qiagen) and post-isolation DNA digestion using Ambion DNA-free DNase Treatment and Removal System according to the manufacturer’s instructions [73]. RNA was quantified on a Nanodrop 1000 spectrophotometer (Thermo Scientific, Wilmington, DE). For all RNAs, cDNA was synthesized using the Superscript VILO cDNA synthesis kit, and SYBR Green RT-qPCR was performed exactly as described [73] for human IFN-α, A3A, A3G, CD317, and CD169 as well as for macaque IFN-α, IFN-β, A3A, A3G, CD169, β7, and MAdCAM-1. Primer efficiency was determined prior to testing mRNA expression in samples. Data were analyzed by the ΔΔCt method. The cell control was RPL19 for human samples and GAPDH for macaque samples. The comparison control was untreated sample from the matched donor for *in vitro* experiments and sample from a single donor (same for all comparisons) for *in vivo* experiments. The fold difference (2^-03940394Ct^) is reported. Primer sequences are provided in S1 Table.

### Statistics

*In vitro* data were analyzed using non-parametric tests with a multiple comparison correction post-test. HIV pulsing experiments included many donors but some donors for which not all conditions could be set up due to limited cell numbers, so the Kruskal Wallis test was used with Dunns correction. In HIV infection experiments, all conditions were set up for each donor so the Friedman test was used with Dunns correction. In order to identify trends at the α < 0.1 level in the *in vitro* studies, Wilcoxon Signed Rank test was performed for datasets with a Friedman *P* < 0.10. Flow cytometry and RT-qPCR from DC and DC-T cell assays were analyzed with Friedman test and Dunns correction. Everywhere multiple comparison correction was used, all comparisons were made except as noted in the figure legend. Wilcoxon Signed Rank test was used for binary comparisons within the *in vitro* datasets (e.g. DC vs. DC-T cell infections from the same donors, pre vs. post HIV pulse flow cytometry data, p24 in single vs. conjugated T cells) as well as for flow cytometry and RT-qPCR data from the same macaques treated with PICLC vs. PBS (and pre vs. post PICLC). Spearman correlation coefficient was used to identify correlations between parameters (e.g. viral load and gene expression). Macaque infection by treatment group was evaluated with two-sided Fisher’s exact test. Viral loads were compared between treatment/virus groups with Mann Whitney test. RT-qPCR data from SIV-infected macaques were analyzed with Friedman and Dunns. *P* values are reported for *P* ≤ 0.1 and were considered significant if *P* < 0.05.

## Results

### Effect of dsRNAs on HIV infection in DC-T co-cultures is contingent on the timing and quality of DC maturation

**Fig 1:**
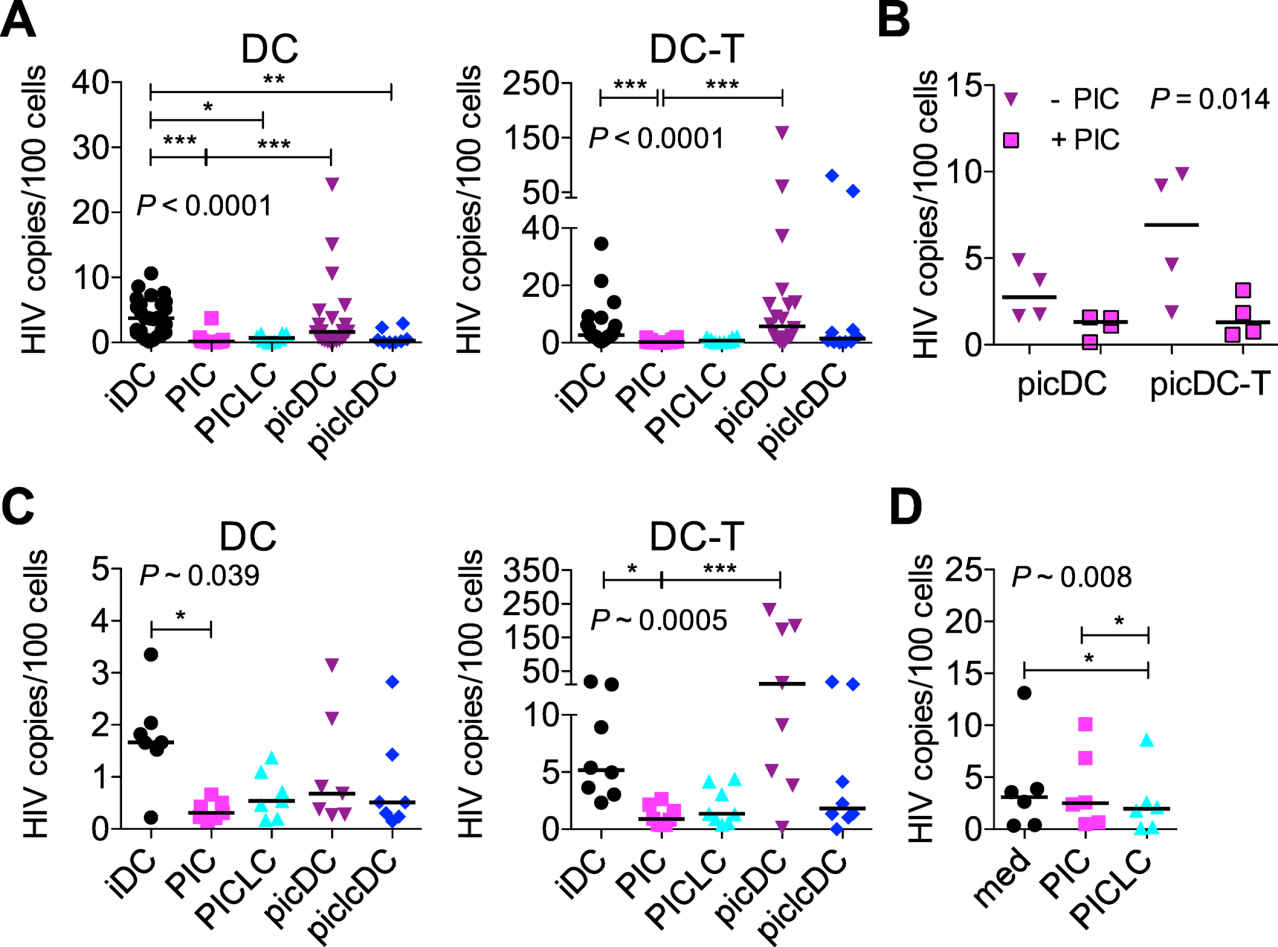
Synthetic dsRNAs block HIV replication in DC-T cell mixtures dependent on timing of DC stimulation and virus capture. Immature DCs (iDCs) were exposed to 10 μg/ml PIC or PICLC for 48 hours to produce picDCs and piclcDCs, respectively, or were maintained as iDCs by 48 hours of culture in medium. (A) iDCs were pulsed with HIV, washed, and re-cultured in the presence of medium (iDC) or 10 μg/ml PIC (PIC) or PICLC (PICLC). picDCs and piclcDCs were similarly pulsed and re-cultured in medium (picDC, piclcDC). After 7 days, the cells were lysed, and HIV DNA was measured by *gag* qPCR (left). Pulsed DCs were cultured with autologous CD4^+^ T cells for 7 days before HIV DNA was measured (right). iDC-T cell co-cultures were left in medium or had PIC/PICLC added as for iDCs alone. For DCs and DC-T cell co-cultures, ≥9 donors are shown with the medians. (B) Responsiveness of picDCs and picDC-T cell co-cultures to exogenous PIC was determined by re-culturing pulsed picDCs (picDC) or picDCs and autologous CD4^+^ T cells (picDC-T) in the presence of 10 μg/ml PIC (+ PIC) vs. medium (- PIC). Four donors and the medians are shown. (C) iDCs (in the presence/absence of 10 μg/ml PIC or PICLC), picDCs, and piclcDCs were infected directly in the plates (not pre-pulsed) with HIV in the absence (left) or presence (right) of autologous CD4^+^ T cells, and HIV DNA was measured in cell lysates after 7 days (7–8 donors and the median). (D) CD4^+^ T cells were infected (not pulsed) with HIV in the presence of 50 U/ml IL-2 and the absence (med) or presence of 10 μg/ml PIC or PICLC before HIV DNA was measured in cell lysates after 7 days (6–8 donors and the median). Statistical analyses that derived the P values shown on the panels were the Kruskal Wallis test in (A) and the Friedman test in (B-D). In all cases, the Dunns test was used for pairwise comparisons, shown as asterisks. All Dunns comparisons were made except in the following cases: In (A) and (C), we did not compare PIC vs. piclcDC or PICLC vs. picDC. In (B), we did not compare picDC-PIC vs. picDC-T+PIC or picDC+PIC vs. picDC-T-PIC. *P<0.05, **P<0.01, ***P<0.001.

We have shown that PIC blocks HIV infection in DCs *in vitro* [32]. PICLC continues to be developed for use *in vivo*, yet little information exists on its impact on *in vitro* DC and DC-T cell biology to help guide development. Upon demonstrating that PICLC has a similar size to PIC (S1 Fig), a known determinant of the downstream response [52], we asked how the timing and quality of DC maturation by PICLC (vs. PIC) would impact HIV replication in DCs and the DC-T cell milieu. We generated picDCs and piclcDCs by maturing iDCs with 10 μg/ml of PIC and PICLC, respectively. This dose of PIC elicited comparable DC responses to those observed using 25 μg/ml for picDCs in our earlier study [32]. We then analyzed the mature DCs’ susceptibility to HIV infection and their ability to fuel HIV infection in DC-T cell co-cultures. In both DCs and DC-T cell mixtures, the time of maturation in the presence of dsRNA impacted the HIV infection outcome (Fig 1A). HIV replication was significantly restricted when either PIC or PICLC was added to virus-bearing iDCs or when piclcDCs were pulsed with HIV (Fig 1A, left). In contrast, picDCs matured before pulsing did not significantly restrict virus replication and in some donors, HIV replicated better in these cells than in the iDCs. HIV replicated significantly better in picDCs than in iDCs with PIC added. Notably, the same was not true for piclcDCs in which HIV infection was restricted.

In virus-pulsed DC-T cell co-cultures, HIV replicated to similar levels as in pulsed iDCs (Fig 1A right; *P* > 0.1 iDCs vs. iDC-T). HIV replication in the DC-T cell co-cultures mirrored that in the DCs alone. Addition of both PIC and PICLC significantly reduced HIV infection in virus-pulsed DC-T cell co-cultures, and HIV replication was not reduced and was sometimes higher in mixtures of picDCs (but not piclcDCs) with T cells. HIV replication in picDC-T cell (but not piclcDC-T cell) co-cultures was also significantly higher than when the matched dsRNA was added to iDC-T cell co-cultures. We confirmed that pre-maturation of DCs with PIC did not render them refractory to the antiviral effects of post-pulsing PIC (Fig 1B). Thus, both PIC and PICLC exert potent antiviral activity in DCs and DC-T cell co-cultures when added after HIV capture, and pre-maturation with PICLC suppresses HIV replication in DCs and DC-T cell co-cultures unlike pre-maturation with PIC.

Since HIV infection outcomes in DC-T cell co-cultures paralleled those in DCs, we wanted to determine if direct effects of dsRNAs on T cells, which express MDA-5, contributed. Thus, we directly infected T cells alongside cultures of DCs and DC-T cell mixtures, without pre-pulsing the DCs (Fig 1C and 1D). This system can less clearly define the role of DCs in HIV infection but more closely resembles the scenario *in vivo*. In CD4^+^ T cells alone, PICLC, but not PIC, significantly reduced HIV replication; however, the magnitude of the reduction was small (Fig 1D).

Addition of HIV to iDC-T cell co-cultures facilitated higher levels of viral replication than those observed in similarly infected iDCs (*P* = 0.016, Fig 1C). dsRNAs mediated comparable (but less pronounced) effects on HIV replication in these DCs and DC-T cell co-cultures as in cultures containing pulsed DCs. picDCs appeared to favor even greater magnitude HIV replication in the infection co-cultures from some donors than in the pulsed co-cultures. Although this observation was not significant, picDCs promoted significantly greater HIV replication than when PIC was added to the co-cultures.

### dsRNAs promote changes in DCs associated with HIV uptake while inducing an innate antiviral state

**Fig 2:**
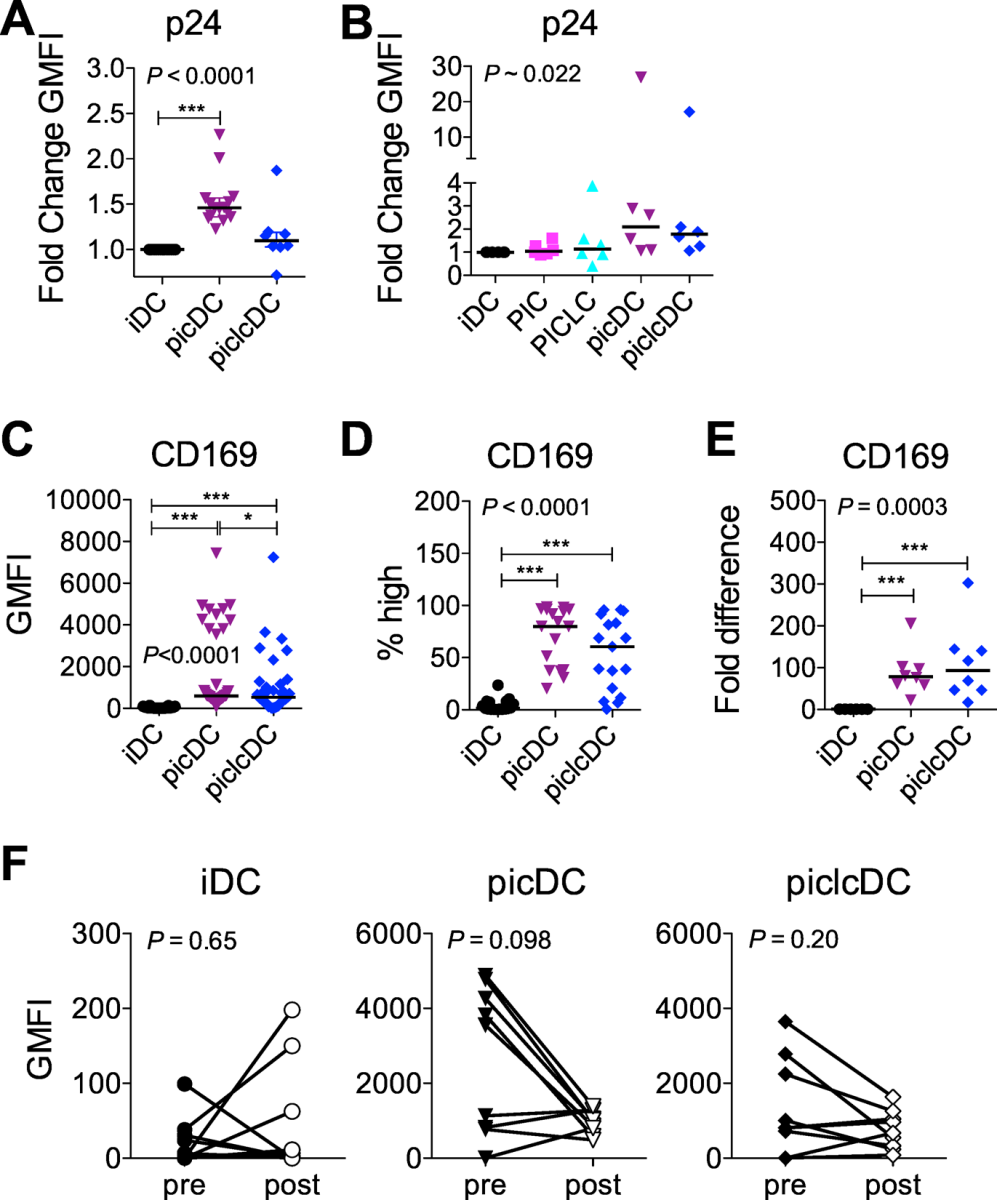
Role of HIV capture by CD169 in dsRNA-mediated effects on HIV infection in DCs and DC-T cell co-cultures. HIV p24 expression was analyzed in DCs after pulsing (A) immediately (8–15 donors, median) or (B) after 24 hours of culture in media or in 10 μg/ml PIC or PICLC (6 donors, median). In a separate set of donors, CD169 surface expression was evaluated by flow cytometry as the (C) geometric mean fluorescence intensity (GMFI) on total DCs (17 donors, median) and (D) the percent of CD169^high^ DCs (17 donors, median). (E) CD169 mRNA expression was evaluated in DCs by RT-qPCR (8 donors, median). (F) The GMFI of CD169 on DCs was compared immediately before and after HIV pulsing (9 donors). In (A-E), statistical analyses that derived the P values shown on the panels were performed using the Friedman test in with post-tests performed using Dunns (significance shown by asterisks). In (B), Dunns post-test did not include PIC vs. piclcDC or PICLC vs. picDC comparisons. In (F) analyses were done using Wilcoxon Signed Rank test. *P<0.05, ** *P*<0.01, *** *P*<0.001.

To dissect the effects of PIC and PICLC on HIV infection in the pulsed DC conditions, we first determined their impact on virus capture by DCs and resulting effects on expression of potential HIV capture and infection molecules. Measuring the amount of p24 associated with pulsed DCs revealed that both picDCs and piclcDCs tended to capture more HIV than iDCs, and this was significant for picDCs (Fig 2A). Addition of PIC or PICLC to virus-loaded iDCs did not impact the retention of p24 over time, but more p24 tended to remain associated with re-cultured picDCs and piclcDCs than iDCs 24 hours post-pulse (Fig 2B, *P* = 0.03 picDC and piclcDC each vs iDC).

CD169 was previously shown to be responsible for increased virus capture by picDCs vs. iDCs [19]. Similarly in our model, DC maturation by PIC increased CD169 surface expression on DCs, the frequency of CD169^high^ cells, and the level of CD169 mRNA (Fig 2C–2E). We measured the decrease in surface expression of CD169, which is suggestive of receptor internalization or usage, and found that on iDCs, which expressed low levels of CD169, surface CD169 expression did not reliably decrease upon HIV pulsing. However, surface expression on picDCs did tend to decrease after HIV pulsing and picDCs that expressed the highest levels of CD169 dropped their surface expression of the receptor most strongly upon HIV pulsing though these trends were not significant (Fig 2F). Importantly, CD169 was comparably upregulated on piclcDCs (Fig 2C–2E), which did not capture as much virus as picDCs (Fig 2A) and which did not as strongly decrease CD169 expression after HIV pulsing (Fig 2F). Examining expression of CD209, CD206, and CCR5 following HIV capture revealed that change in surface expression of HIV capture and infection molecules varied considerably between donors and maturation conditions (S2 Fig). piclcDCs significantly reduced expression of surface CD206 after HIV pulsing (S2 Fig), suggesting that CD206 may be utilized for HIV capture on these cells. In general, each molecule’s level of surface expression tended to be associated with the extent of its decreased surface expression upon HIV binding.

**Fig 3:**
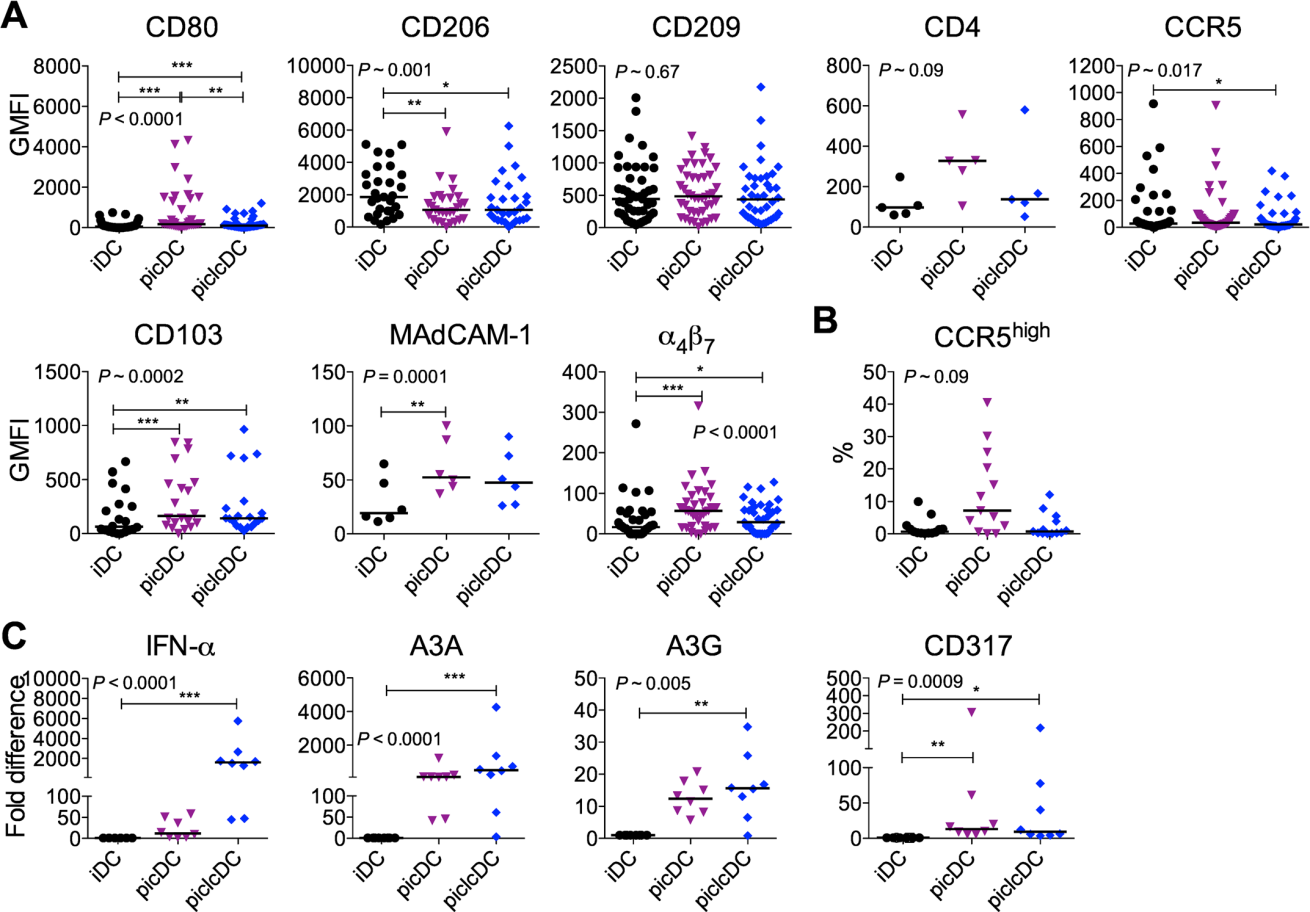
Synthetic dsRNAs induce varying levels of DC maturation, HIV-capture molecules, and antiviral factors. The surface phenotype of DCs generated as in Fig 1 was assessed. (A) The GMFI of the indicated markers was measured on the total DC population (21–43 donors with median except for MAdCAM-1 and CD4 with 5–6 donors). (B) The frequency of CCR5^high^ DCs within the total DC population (41 donors with median). (C) mRNA levels of IFNα, A3A, A3G, and CD317 in DC lysates (8 donors with median). In (A-C), statistical analyses that derived the P values shown on the panels were performed using the Friedman test in with post-tests performed using Dunns (significance shown by asterisks). All Dunns comparisons were performed. *P<0.05, **P<0.01, ***P<0.001.

To further explore how PICLC (vs. PIC) impacted HIV uptake, transfer, and replication, we more extensively defined the DC phenotypes. As previously shown for PIC [32], both long dsRNAs induced partial phenotypic maturation characterized by increased CD80, CD83, CD86, and HLA-DR, decreased CD206, and little change in CD25 (Fig 3A and S3 Fig). However, activation was generally stronger in picDCs than piclcDCs, especially with respect to increases in CD80, CD83, and CD86. Contrasting our previous results showing a mild effect of PIC on CD209 expression [32], herein at a lower dose of dsRNA, maturation had no significant effect on CD209. In addition, analysis of trends suggested that PIC, but not PICLC, may have increased CD4 expression (*P* = 0.063 picDC vs. iDC) and the frequency of CCR5^high^ cells (P = 0.003 picDC vs. iDC, Fig 3B) while PICLC significantly reduced overall CCR5 per cell expression (Fig 3A).

We recently showed that DCs imprinted with a semi-mature mucosal-like phenotype upon retinoic acid (RA) conditioning drive infection in DC-T cell co-cultures in a manner involving MAdCAM-1, the natural ligand for the gut homing integrin, α_4_β_7_ [24]. To investigate if the trend for increased infection in picDC-T cell co-cultures was similarly associated with a mucosal DC phenotype, we measured the expression on dsRNA-matured DCs of MAdCAM-1, α_4_β_7_, and another mucosal homing integrin, CD103. picDCs and piclcDCs exhibited the mucosal-like phenotype, having increased expression of CD103, MAdCAM-1, and α_4_β_7_ (Fig 3A). As for the maturation markers, the difference between iDCs and dsRNA-matured DCs was greater for picDCs than piclcDCs. Thus, maturation by both long dsRNAs imparted DCs with a surface phenotype promoting HIV capture and DC-T cell transfer, which was more pronounced for PIC than PICLC. PIC, but not PICLC, also induced a phenotype promoting DC infection.

Since PIC triggers antiviral responses in DCs [32], we investigated if PICLC differentially mediated antiviral effects alongside the differences in maturation by quantifying the induction of the type I IFN pathway. As expected, DC maturation by these dsRNAs induced expression of type I IFN and downstream IFN-inducible antiviral host factors including A3A, A3G, and CD317/tetherin (Fig 3C) [32, 74, 75]. In contrast to the effects on maturation, PICLC was the stronger inducer of IFN-α though this was less evident at the level of IFN-stimulated genes.

**Fig 4:**
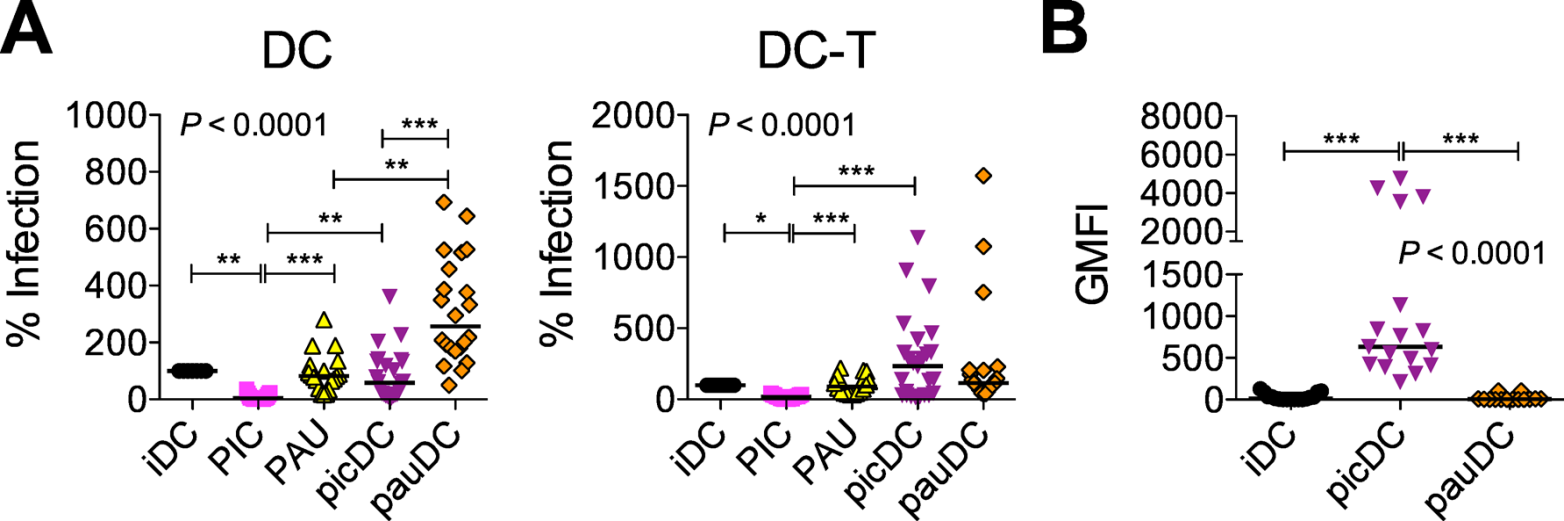
PAU promotes CD169-independent HIV replication in DCs and DC-T cell mixtures. (A) picDCs and pauDCs were generated and pulsed with HIV and 7 day cultures were established as described for picDCs and piclcDCs in Fig 1. Results from HIV gag qPCR are shown for each condition as a percent of the infection in the iDC (left) or iDC-T cell (right) control. More than 9 donors are shown with the median for each condition. (B) GMFI of CD169 is shown on the differently matured vs. immature DCs for 10 donors (shown with the median). In (A-C), the statistical analyses used the Friedman test with Dunns post-test. In (A), Dunns post-test did not include PIC vs. pauDC or PAU vs. picDC comparisons. *P<0.05, **P<0.01, ***P<0.001.

Since PIC and PICLC are both ligands for TLR3 and MDA-5, it is possible that differential effects of the dsRNAs could be due to triggering DCs through different PRRs despite the similar size of the molecules [52, 53, 55]. Thus we also examined the effect of the TLR3-only agonist PAU, a dsRNA smaller than PIC or PICLC (S1 Fig) in this system. Interestingly, PAU did not inhibit HIV replication in iDCs or iDC-T cell mixtures, and HIV replicated better in pauDCs and in some donors also replicated better in pauDC-T cell co-cultures than in paired cultures with iDCs (Fig 4A). Notably however, PAU did not upregulate CD169 expression (Fig 4B). PAU also did not phenotypically mature the DCs (no change in CD80, CD83, CD86, CD25, HLA-DR, or CD206); did not induce the mucosal phenotype (no change in α_4_β_7_ or CD103); did not affect expression of CD4 or CCR5; and did not induce A3A or A3G (S3 Fig).

### dsRNA-matured DCs promote HIV replication in conjugated T cells

**Fig 5:**
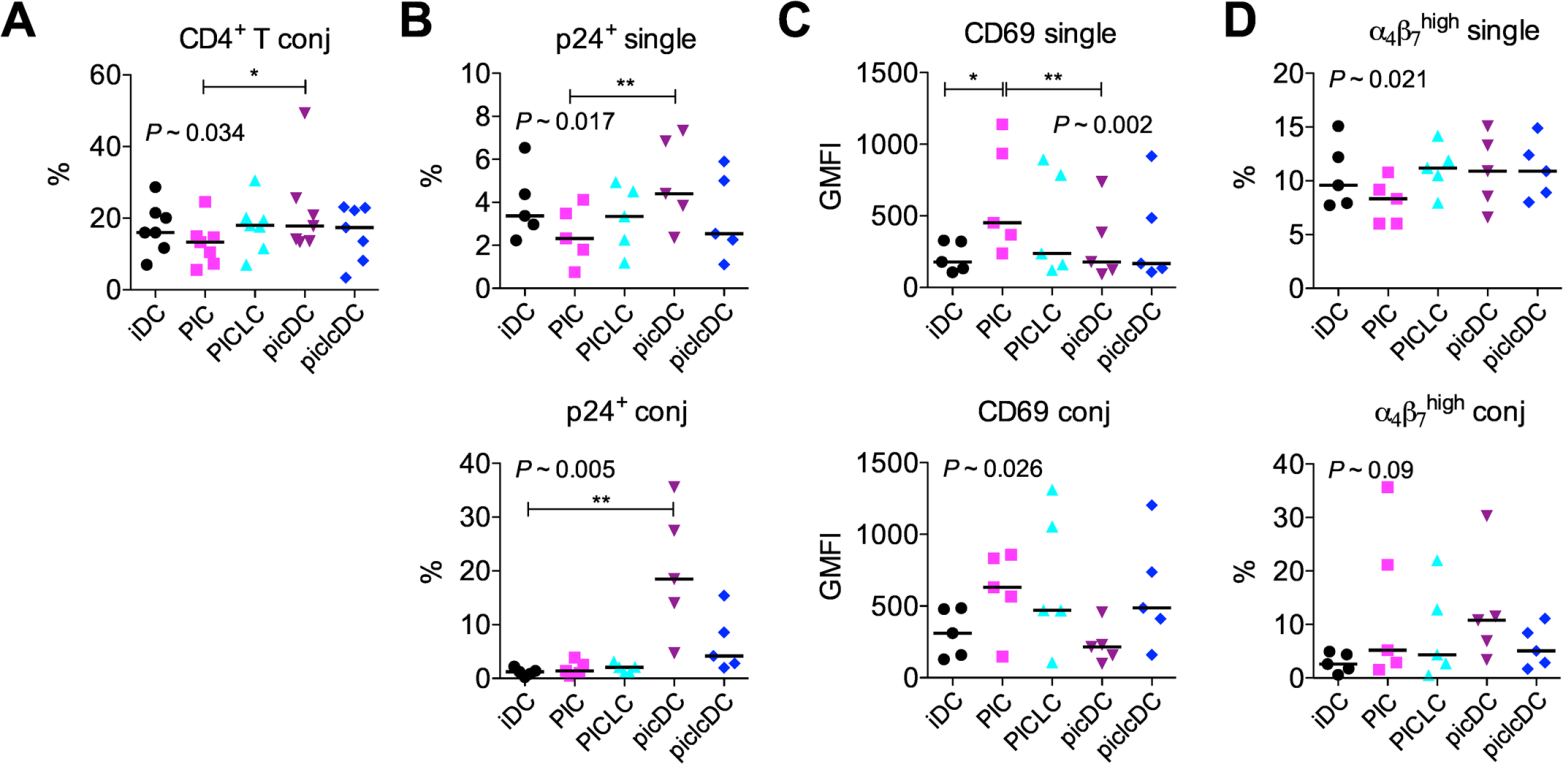
dsRNAs mediate changes in HIV location and T cell phenotype within co-cultures. HIV-pulsed DCs were co-cultured with autologous CD4^+^ T cells in the presence or absence of dsRNAs as in Fig 1. After 24 hours, cells were collected, surface stained, and intracellularly stained for p24. (A) Conjugate frequency within DC-T cell co-cultures was defined as the proportion of live CD3^+^CD4^+^ large cells in the co-cultures (see Methods). (B) Frequency of p24^+^ cells within the populations of free T cells (single) and conjugated T cells (conj). (C) CD69 GMFI and (D) the percentage of α_4_β_7_^high^CD45RO^+^ CD4^+^ T cells were monitored within the single and conjugated T cell populations. For (A-D), 5 donors and the medians are shown, and the Friedman test with Dunns post-test was used to analyze the data. Dunns post-test excluded comparisons of PIC vs. piclcDC and PICLC vs. picDC. *P<0.05, **P<0.01.

To determine how the aforementioned effects of dsRNAs on DCs impacted co-cultured CD4^+^ T cells and HIV therein, we measured the effects of dsRNAs and dsRNA-matured DCs on (1) DC-T cell conjugate formation, (2) p24 levels in the single and conjugated T cells, and (3) the T cell phenotype 24 hours after DC-T cell co-culture. PICLC and piclcDCs had no impact on conjugate frequency; however, the frequency of DC-T cell conjugates was significantly greater in the presence of picDCs than when PIC was added to iDC-T cell co-cultures (Fig 5A). The highest median level of p24^+^ single T cells and conjugates was also found in co-cultures containing picDCs. In the single T cells, this was significantly greater than when PIC was added to the co-cultures and in conjugates, it was significantly greater than in co-cultures with iDCs (Fig 5B). Although the difference was less pronounced than with the picDCs, piclcDC-containing co-cultures also tended to have more p24^+^ conjugated cells than co-cultures containing iDCs (*P* = 0.063 vs. iDC-T). Addition of PIC (but not picDCs) significantly increased CD69 expression (Fig 5C) and tended to decrease the frequency of α_4_β_7_^high^CD45RO^+^ memory single T cells (*P* = 0.011 vs. iDC-T, Fig 5D). In contrast, conjugates from picDC-T cell co-cultures, which contained the highest p24 levels, expressed low levels of CD69 and high frequencies of α_4_β_7_^high^CD45RO^+^ cells. Thus, the timing and quality of DC maturation governed DC-T cell communication, T cell activation, and the location and levels of HIV replication at this early time point. The levels of HIV seen in T cells 24 hours after initiating the co-culture were not predicted by the effects of DC-mediated T cell activation or induction of an HIV-susceptible phenotype but did predict the levels of HIV replication seen by qPCR after 7 days. Paralleling what was observed in the DCs, when PIC and PICLC were added to co-cultures, they significantly increased the transcript levels of IFN-α and A3G, with PICLC the more potent antiviral stimulus (S4 Fig).

To validate that the effects of dsRNAs observed in the reconstructed DC-T cell system were relevant in a mixed leukocyte population of *in vivo*-derived cells, we cultured unfractionated human PBMCs overnight with PIC or PICLC vs. media and measured mDC and CD4^+^ T cell activation after 24 hours. In agreement with the results from the DC-T cell model, expression of CD169, α_4_β_7_, and CD80 by CD11c^high^Lin^-^HLA-DR^+^ mDCs and CD69 expression by CD4^+^ T cells were more effectively upregulated by PIC than PICLC (S5 Fig). PICLC had little, if any, effect on CD169 expression in the mixed cell population. Taken together, these *in vitro* results demonstrate that PIC is a more potent DC maturation and T cell activation stimulus than PICLC *in vitro* while PICLC is a more potent activator of the IFN antiviral pathway. Both dsRNAs can exert powerful antiviral responses to block HIV infection when introduced at the right time.

### PICLC activates macaque DCs and T cells *in vivo*

**Fig 6:**
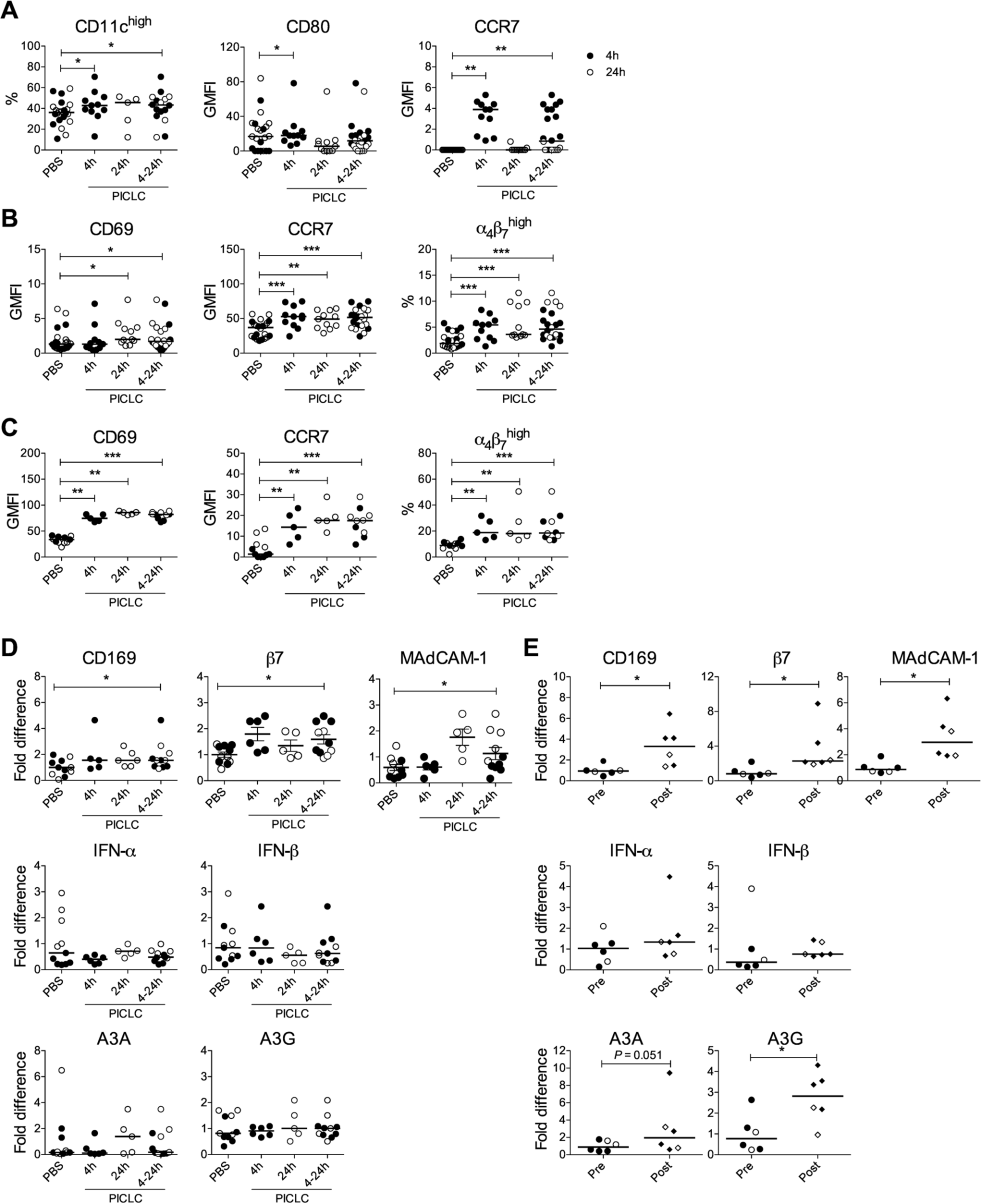
Rectally applied PICLC induces rapid local and systemic immune changes. Macaques (n = 11) were bled 4 hours (4h) and 24h after rectal PBS vs. PICLC application. Either 4h (n = 6) or 24h (n = 5) after receiving treatment, rectal biopsies were also collected. (A) Blood mDCs were characterized at the indicated times post-treatment by their frequency (%Lin^-^HLA-DR^+^CD11c^high^) and expression of CD80 and CCR7. (B) Blood and (C) rectal CD4^+^ T cells were characterized by their expression of CD69 and CCR7 and the frequency of α_4_β_7_^high^CD95^+^ cells. (D) mRNA levels of the markers shown were measured in rectal tissue. (E) In a separate group of macaques biopsied 5 weeks before (Pre) and 24 hours after (Post) a single rectal application of 2 mg (filled symbols) or 4 mg (open symbols) PICLC, mRNA levels of the markers from (D) were measured in cells isolated from rectal tissue. In (A-E), statistical analyses using the Wilcoxon Signed Rank test compared the post-PICLC time points with control post-PBS time points in each animal. *P<0.05, **P<0.01, ***P<0.001.

Knowing that PICLC has differential effects on moDC biology that influence HIV replication in DCs and DC-T cell mixtures *in vitro*, and that PICLC is being used clinically, we were interested to examine its effects *in vivo* on mucosal DC-T cell biology and HIV transmission. Adding to what is known about secretion of antiviral cytokines by macaque DCs treated with PICLC [30], we found that like human mDCs, macaque mDCs upregulated CD169 in response to *in vitro* PIC but not PICLC treatment (S5 Fig). We then administered PICLC rectally to macaques and monitored cell subsets in blood and rectal mucosa 4–24 hours post-exposure in comparison with prior placebo (PBS) treatment of the same animals (S6 Fig). Rectal PICLC increased the frequency and activation state (CD80 and CCR7 expression) of circulating CD11c^high^ mDCs (Fig 6A) and bystander CD123^+^ plasmacytoid DCs (pDCs, S7 Fig) within 4 hours of exposure. mDC (but not pDC) activation returned to baseline by 24 hours. PICLC also activated CD4^+^ T cells (CD69 and CCR7 expression) and increased the frequency of α_4_β_7_^high^CD4^+^CD95^+^ memory T cells in peripheral blood and rectal mucosa within 4 hours of exposure, for up to at least 24 hours (Fig 6B and 6C). Importantly, despite the phenotypic activation of peripheral blood cells, no changes in plasma Th1 cytokines or chemokines were detected (S1 Methods, S8 Fig).

We examined expression of other genes within the rectal tissue by RT-qPCR. Paralleling the *in vitro* results, PICLC increased CD169, β7 and MAdCAM-1 (Fig 6D). However, we could not detect any impact of rectal PICLC on the type I IFN pathway, measured by transcription of IFN-α, IFN-β, A3A, and A3G. To determine if this was a true lack of IFN induction in response to mucosal PICLC *in vivo* or if it could be a dose effect or masked by the low frequency of responding cells in the tissue, we measured expression of these genes in another cohort of macaques treated with 2mg or 4mg single doses of PICLC 24 hours earlier (S6 Fig). We found larger increases in CD169, β7, and MAdCAM-1 as well as an increase in A3G and a marginal increase in A3A that were not detected in the first study (Fig 6E). IFN-α and IFN-β expression tended to be higher 24 hours after PICLC treatment, but this was not significant in the small number of animals that were available to be tested, and no samples were collected from earlier time points (e.g. 4 hours) when these mRNAs might have been more abundant. We did not test for IFN proteins in rectal swab fluid from these animals, but protein levels were shown to parallel mRNA levels in previous work [76].

### Rectal PICLC tends to decrease SIV acquisition and increases SIVΔNef replication

**Fig 7:**
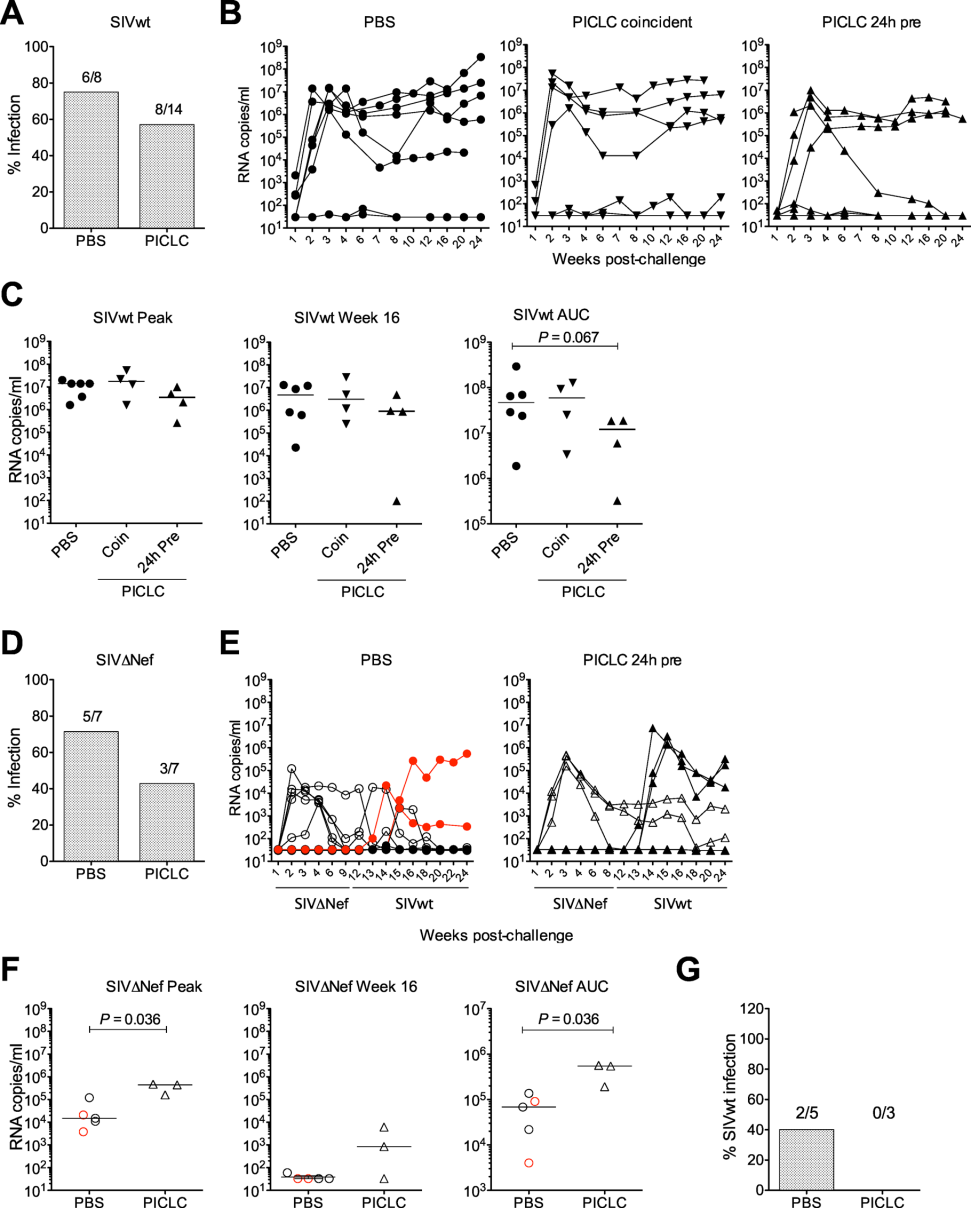
Rectal PICLC modestly decreases SIV transmission but increases SIVΔNef replication in infected animals and promotes the vaccine effect. (A) Macaques were rectally challenged with 3000 TCID_50_ SIVmac239 (SIVwt) coincident with (n = 7, “Coincident”) or 24 hours after (n = 7, “24h pre”) rectal PICLC or 24 hours after rectal PBS (n = 8). The fraction of PBS vs. PICLC-treated macaques that became infected is shown as a percent, and the number of animals infected is above each bar. (B) SIV RNA copies/ml were measured over time in each animal shown in (A). (C) Plasma viremia in infected animals in each group is shown at peak (highest observed viremia, 2–4 weeks post-challenge in all macaques, left) and 16 weeks post-challenge (middle), and as the area under the curve (AUC) of viremia over the whole observation period (right). The timing of PICLC administration is denoted by the symbols used in (B). (D) Macaques were rectally challenged with 3000 TCID_50_ SIVmac239ΔNef (SIVΔNef) 24 hours after rectal PICLC (n = 7) or PBS (n = 7). 12 weeks after SIVΔNef challenge, all animals were rectally challenged with 3000 TCID_50_ SIVwt. The fraction of PBS vs. PICLC-treated animals that became infected with SIVΔNef is shown as a percent, and the number of SIVΔNef-infected macaques is above each bar. (E) SIV RNA copies/ml of SIVΔNef (open symbols) and SIVwt (filled symbols) were measured over time in each animal. The two SIVΔNef-infected macaques not protected from SIVwt are shown in red. (F) SIVΔNef plasma viremia in each group is shown at peak (2–4 weeks post-challenge, left) and 16wks post-challenge (4 weeks post-SIVwt challenge, middle), and as AUC (right). The two macaques not protected from SIVwt are shown in red. (G) Fraction of SIVΔNef-infected animals that subsequently also became infected with SIVwt is shown by treatment group. In (C) and (F), P values were derived from Mann Whitney test comparisons of the control group with each of the treated groups.

Paralleling the *in vitro* findings, rectal PICLC induced a mixture of responses locally and systemically that on their own can drive or inhibit HIV in the DC-T cell milieu. To determine which effects would prevail in determining immunodeficiency virus transmission across the rectal mucosa, we treated naïve macaques with PICLC or placebo (PBS) and challenged them with SIVwt (S9 Fig). Since the timing of DC maturation directed the *in vitro* infection results, treating with PICLC after SIV challenge would have most closely paralleled the best inhibition of HIV we saw *in vitro*. However, this approach seemed less viable *in vivo* where virus rapidly disseminates from the site of challenge to invade multiple tissue layers. Instead, we compared application of PICLC coincident with SIVwt vs. 24 hours earlier. Pre-treatment with PICLC in mice has been shown to effectively inhibit influenza infection [77]. Of the 8 control animals, 6 became productively infected with SIVwt (75% infection rate) after a single high dose challenge (Fig 7A). PICLC application reduced this to 4 of 7 (57% infection rate) in both groups (coincident and 24h pre, 8 of 14 total), but this was not significant (*P* = 0.65 for each and when test groups were pooled). Two macaques in the coincident group, 1 in the 24h pre group, and 1 in the PBS group experienced very low-level infection with plasma viremia nearing 100 copies/ml on a few sporadic time points, but this did not meet our criteria for a productive infection (see Methods and Table 1) [70, 71]. Rectal PICLC exerted no significant influence on SIVwt viral replication in the periphery during either acute or chronic infection although macaques that became infected following the PICLC 24 hour pre-treatment tended to have less SIVwt circulating in blood over the course of infection than untreated macaques (Fig 7B and 7C). Rectal PICLC had no significant impact on peripheral CD4^+^ T cell depletion in infected animals (Table 1). Of note, measurement of plasma IFN-α levels revealed a tendency for plasma IFN-α to decrease rather than increase following co-administration of rectal PICLC and SIVwt. Plasma IFN-α levels did not correlate with infection outcome (S1 Methods, S10 Fig). Rectal fluid and tissue levels of IFN-α could not be measured around the time of challenge.

To discern whether the effects of PICLC on DC and T cell activation would be revealed as differences in transmission of SIVwt vs. SIVΔNef (viruses with distinct activation requirements for replication), we challenged another group of macaques rectally with a single high dose SIVΔNef challenge 24 hours after rectal PICLC vs. PBS dosing (S8 Fig). We used the 24 hour timing because pre-treatment with PICLC gave similar results as coincident exposure, PICLC reduced SIVΔNef infection to 3 of 7 (43% infection rate) vs. 5 of 7 controls (71% infection rate), which was similar to what we observed in the case of SIVwt challenge and was not significantly different (*P* = 0.59) (Fig 7D and 7E). Notably, among animals that became infected with SIVΔNef, those treated with PICLC exhibited a peak viral load that was significantly higher than that of the controls (Fig 7F left). Two of the 3 PICLC-treated SIVΔNef-infected macaques, but none of the 5 controls, also exhibited persistent SIVΔNef viral replication (>100 copies/ml) later than week 12 (Fig 7E and 7F middle), and by area under the curve (AUC) analysis, SIVΔNef-infected animals that received PICLC had significantly higher viral loads than controls overall (Fig 7F right). Four of the 5 SIVΔNef-infected PBS-treated macaques actually completely controlled replication of the virus within 6 weeks (Fig 7E).

SIVΔNef replication correlates with protection from pathogenic SIV infection [70, 78]. To determine if the increased SIVΔNef replication in PICLC-treated macaques improved the protective effect against SIVwt, we re-challenged all the animals rectally with SIVwt 12 weeks after SIVΔNef challenge (S9 Fig). We recently showed that SIVΔNef infection by the rectal route completely protects against rectal SIVwt acquisition 15 weeks post-SIVΔNef [79], and we selected a slightly earlier time point in order to see if PICLC might decrease breakthrough SIVwt transmission. In the PICLC group, the 4 animals that did not become infected with SIVΔNef became infected with SIVwt, and virus replicated normally (Fig 7E right). All 3 PICLC-treated SIVΔNef-infected macaques were protected from SIVwt (Fig 7G) though 2 of them experienced increased SIVΔNef replication following SIVwt challenge (Fig 7E right). Neither of the SIVΔNef-uninfected macaques in the control group became infected with SIVwt; one of these manifested a blip in SIVΔNef viremia 2 weeks post-SIVwt challenge, suggesting the possibility of a highly controlled low-level SIVΔNef infection in this animal though no SIV gag DNA was ever detected in PBMCs from this animal. Unlike the 3 PICLC-treated SIVΔNef-infected macaques that were completely protected from SIVwt, 2 of the 5 PBS-treated macaques infected with SIVΔNef were not protected from SIVwt (Fig 7G). One of these did not control SIVΔNef viremia; the other had the lowest (<10^4^ copies/ml) and latest (week 4) SIVΔNef peak viremia (Fig 7E and 7F). In both of these animals, SIVwt viremia was truncated, indicating an effect of SIVΔNef in modulating the SIVwt infection post-acquisition (Fig 7E left).

### Rectal CD169 and β7 expression differently correlate with viral load during SIV infection

**Fig 8:**
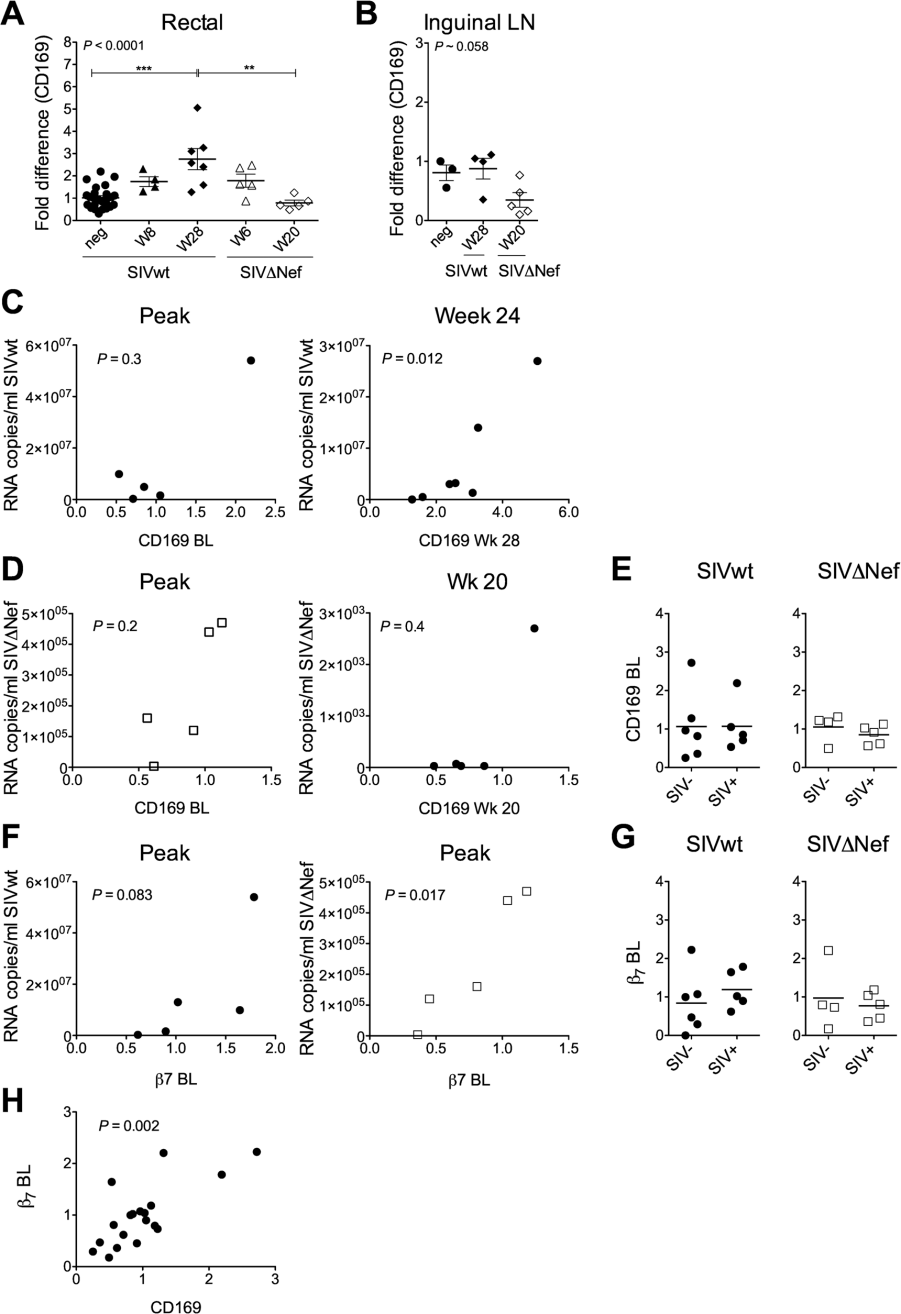
Rectal CD169 and β_7_ expression correlate with systemic virus replication but do not predict infection. CD169 mRNA levels were measured in (A) rectal tissues and (B) inguinal LNs from macaques infected with SIVwt and SIVΔNef at different times post-infection and in uninfected macaques (baseline of the infected and other macaques that did not become infected within the study). Correlations between rectal CD169 level and viral replication in (C) SIVwt and (D) SIVΔNef-infected macaques are shown. (E) Relationship between baseline rectal CD169 expression and infection outcome for SIVwt and SIVΔNef. (F) Correlation between baseline rectal β_7_ level and peak viral loads in SIVwt and SIVΔNef-infected macaques is shown. (G) Relationship between baseline rectal β_7_ level and infection outcome. (H) Correlation between rectal CD169 and β_7_ levels at baseline for all animals challenged with SIVwt and SIVΔNef. In (A-H), samples from all challenged macaques were not available at every time point. In (A-B), statistical analyses used the Kruskal Wallis test and Dunns post-test. In (A), Dunns comparisons not made were SIVwt W8 vs. SIVΔNef W20 and SIVwt W28 vs. SIVΔNef W6. In (C), (D), (F), and (H), Spearman correlation coefficient was determined. *P<0.05, **P<0.01, ***P<0.001.

In addition to capturing HIV on DCs, CD169 is increased during inflammatory processes and is upregulated on monocytes *in vivo* during HIV [80, 81] and SIV [82] infection and correlates with viral load [80]. Although PICLC-induced changes *in vivo*, including an increase in CD169 expression, were not associated with increased transmission of either SIVwt or SIVΔNef, we sought to determine whether CD169 expression was differentially affected by SIVwt and SIVΔNef infection, independent of PICLC treatment. Monitoring CD169 expression in rectal tissues from macaques infected with SIVwt vs. SIVΔNef revealed that while expression tended to be higher within 6–8 weeks following both SIVwt and SIVΔNef infections (and was not different between SIVwt and SIVΔNef), expression continued to increase during chronic infection only in SIVwt-infected animals (Fig 8A). By contrast in macaques infected with SIVΔNef, CD169 expression in rectal tissue during the chronic phase was as low as in uninfected macaques. In inguinal LNs from these animals, CD169 expression was even lower in chronic SIVΔNef infection than in the absence of infection while the elevation in CD169 expression during chronic SIVwt infection was less pronounced (Fig 8B). As expected based on the transmission data, rectal tissue expression of CD169 at baseline did not correlate with peak viremia in either SIVwt or SIVΔNef-infected macaques (Fig 8C and 8D left), and baseline CD169 expression did not predict SIV acquisition (Fig 8E). However, late in SIVwt infection, CD169 levels correlated with plasma viral load (Fig 8C and 8D, right). This was not the case for SIVΔNef infection, but most of the animals controlled infection below the limit of detection at this time, and the macaque with the highest CD169 level did have the highest SIVΔNef viral load (Fig 8D, right).

Having previously shown that the frequency of blood memory CD4^+^ T cells expressing high levels of α_4_β_7_ both predicted SIV susceptibility and correlated with the frequency of these cells in rectum [83], we also examined expression of β_7_ in the rectal tissue. In contrast to CD169, baseline tissue β_7_ expression correlated with peak SIVΔNef viral load and tended to correlate with peak SIVwt viral load. Although baseline expression did not significantly predict acquisition of either virus, there was a trend towards higher β_7_ expression in the macaques that became infected with SIVwt (Fig 7G). Of note, baseline expression of β_7_ and CD169 in rectal tissue was highly correlated (Fig 7H).

## Discussion

Although innate immunity and accompanying inflammation are the first line of defense against viral exposure at mucosal surfaces, the detrimental association between mucosal inflammation and HIV susceptibility has been largely thought to outweigh any potential protective effects of a heightened DC-driven innate response [7, 84]. However, recent studies show that early, appropriately timed IFN-α treatment of SIV-exposed macaques prevents systemic infection [85], innate responses are present in highly exposed HIV seronegative subjects [86], and DCs from HIV-infected elite controllers have increased expression of ISGs [87]. These results are recalibrating the thinking around immune activation and setting the stage for further exploration into how to properly harness DCs and the type I IFN response as a component of approaches for HIV prevention and therapy. PICLC is being developed for clinical use in several arenas [37] including as a latency-reversing drug along the lines of TLR7 agonists [47, 88–90], and PIC is a superior TLR-based adjuvant for eliciting HIV-specific T cell responses [25]. Yet PICLC’s direct impact on HIV transmission *in vivo* has until now not been determined, and no studies have compared PICLC with PIC head to head *in vitro*. Thus, we sought to achieve two objectives: (1) to use the *in vitro* moDC-CD4^+^ T cell model of mucosal transmission of a CCR5-tropic virus to explore how DC maturation by PICLC might influence the outcome of HIV infection in DCs and DC-T cell co-cultures, and (2) to directly relate these findings to effects of PICLC *in vivo* against rectal SIV transmission. We found that PICLC (1) induced type I IFN responses and DC and T cell activation *in vitro* and *in vivo*; (2) shut down HIV infection in the DC-T cell environment; (3) modestly though non-significantly, reduced SIV acquisition in macaques in the absence of any additional immunogen; and (4) increased SIVΔNef replication in SIVΔNef-infected animals, which may have improved their protection from SIVwt. More broadly, we demonstrated that maturation of DCs with dsRNAs induced a mixture of effects on the DCs’ capacity to capture, become infected by, and replicate HIV, as well as to interact with and activate T cells and induce antiviral responses. The dsRNA used and the timing of stimulation relative to virus exposure governed differential effects on HIV replication. Our results are consistent with previous reports on the timing of DC maturation and type I IFN responses *in vitro* [17, 28] and *in vivo* [85], and underscore the complex biology of DC-driven HIV infection.

When PICLC and PIC were added to DCs or DC-T cell mixtures, both exerted potent restriction to HIV replication. Despite the two dsRNAs having similar lengths, PICLC induced the stronger antiviral response. Only piclcDCs were resistant to HIV infection though they still transferred virus to T cells, albeit less efficiently than picDCs. The divergent results for picDCs vs. piclcDCs (alone and in co-culture with T cells) could be related to (1) the more effective induction of HIV capture and infection molecules on picDCs; (2) the larger amount of virus captured by and replicating in those cells; (3) contributions of *cis* and *trans* transfer in picDC-T cell mixtures; and (4) the greater antiviral impact of PICLC on DC and T cell infection. That less pronounced effects of the dsRNAs were observed in the infection vs. the pulse model may be simply because in the former, virus was not limiting in the culture so any effects on HIV capture and infection of DCs would be muted.

Overall, PIC and PICLC exerted similar effects on DC maturation and HIV infection *in vitro*, but there were some differences that suggest a role for persistence of PICLC as well as potential differences in receptor utilization and downstream signaling. Although PIC can recognize TLR3 and MDA-5 *in vitro* [51, 52, 91], it was reported that PIC-mediated DC maturation was optimal only when both TLR3 and MDA-5 were engaged [92]. Maturation has been shown to require IFN signaling [50, 92, 93], and we also previously showed that blockade of the IFN receptor abrogated PIC-mediated protection from HIV in iDCs [32]. In mice, TLR3 was dispensable while MDA-5 was required for IFN induction by PIC [29, 51, 56]. In our study, PICLC mediated a strong IFN response in DCs and DC-T cell mixtures, even stronger than PIC, and despite the lack of large changes in DC surface phenotype.

To definitively assign roles of TLR3 and MDA-5 in the effects of PIC and PICLC on HIV replication and DC phenotype in our work would require knockdown experiments. These methods are difficult in DCs, especially so when the genes of interest are IFN-related as off target IFN-related effects are often observed [32]. Nonetheless, knockdown can be done in DCs [94] and would clarify these results. Using PAU, which engages TLR3 but not MDA-5, we found that PAU did not induce type I IFN, did not impact HIV replication in iDCs or iDC-T cell co-cultures, and did support HIV replication within DCs and co-cultured T cells when used to pre-mature the cells (pauDCs). This was not likely a dose effect; being that PAU is smaller than PIC/PICLC, 10 μg/ml would have delivered a larger molar quantity of dsRNA. Interestingly, enhanced HIV infection by PAU occurred in the absence of DC activation or any increase in capture/infection molecules, including CD169. Thus, while TRIF-dependent TLR ligands have been implicated similarly in regulating CD169 expression [14, 19], not all TLR3 ligands increase CD169, and additional molecules must be involved in the DC-T cell interplay that drives infection. Fittingly in our experiments, CD169 appeared to be utilized most strongly when it was most expressed while other molecules (e.g. CD206 or CD209) may have been used when they were more highly expressed. Taken together, our results suggest that the IFN response and potentially also utilization of MDA-5 play major roles in HIV restriction by dsRNA *in vitro* in opposition to the phenotypic changes in DCs that facilitate HIV infection and T cell transfer.

The potential for different PRR requirements, and downstream effects *in vitro* and *in vivo* [51], underscore the importance of testing DC activation strategies in animal models. Ultimately, our studies with PICLC in the DC-T cell model supported the *in vivo* findings but additionally revealed *in vitro*/*in vivo* differences. PICLC significantly effected local and systemic changes in DC and T cell biology *in vivo* and induced antiviral responses that slightly reduced both wt and ΔNef SIV infections although the effect, if any, was too small to achieve significance with the number of animals used. The lack of significant effect was not likely due to the PICLC dose, which was based on our previous work in macaques [41, 42, 64] and doses being developed for adjuvant purposes in humans [43], or the double dosing technique, which mirrors a prime boost vaccination strategy [50] and is often utilized for dsRNA delivery [42, 50, 64]. Human volunteers exhibited peak responses approximately 24 hours after single subcutaneous injection [43], mirroring the acute response to mucosally administered PICLC here in macaques. Importantly, the high dose challenge model utilized could have overwhelmed our ability to see a small effect of PICLC. Previous work failed to demonstrate efficacy of intravaginally delivered TLR7/9 ligands against high dose intravaginal SIVmac251 challenge despite inducing IFN-α and other antiviral cytokines [76]. Notably in that study, repeated pre-challenge treatment with either TLR7 or TLR9 ligands increased SIV set point viral load, pointing to the impact of HIV-augmenting effects of DC maturation and innate immune stimulation (including IFN induction). In our study, the antiviral responses appeared weaker *in vivo* than *in vitro* (at the times examined) while PICLC still established an activated immune environment characterized by increased expression of T cell and DC activation markers, CD169, α_4_β_7_ and MAdCAM-1, which encourage DC-driven HIV replication *in vitro* [24].

PICLC likely induced type I IFN production from multiple cell types in the rectal tissue including NK cells [29], but we did not explore the relative contribution of different cell types to effects on SIV infection in this study. It is worthwhile noting that rectal PICLC resulted in phenotypic activation of circulating pDCs, most likely through bystander effects but potentially through engaging MDA-5 within pDCs in the tissue. Activation of pDCs very early in acute infection could lead to IFN-mediated protection, but alternatively, could drive immune activation fueling virus amplification. Importantly, we did not observe an increase in the plasma levels of IFN-α or any Th1 cytokines resulting from rectal PICLC administration, and in fact observed a decrease in plasma IFN-α after rectal PICLC. Together with phenotypic activation of pDCs, these data suggest pDCs may have been recruited to the rectal mucosa where they may have participated locally in protection. Unfortunately, we were unable to take acute mucosal samples from challenged animals, precluding any correlations between specific local immune changes and SIV transmission. Exploring SIV exposure at different times relative to PICLC treatment (e.g. PICLC treatment acutely following SIV challenge [85]) might impact the infection outcome; future studies are needed to address this.

We utilized both pathogenic SIVwt and non-pathogenic SIVΔNef infections to try to tease out roles of mDCs in SIV transmission. DC and T cell activation by PICLC may help to explain the increased SIVΔNef (but not SIVwt) replication in the animals that became infected even though the frequency of SIVΔNef infection was not reduced. Interestingly, immune activation as measured by CD169 expression did not parallel the heightened viral replication in these animals either in blood or LNs. Since SIV Nef counteracts tetherin [95], and differential tetherin-mediated restriction could have resulted in differences between SIVwt and SIVΔNef replication after PICLC treatment. However, we expected viremia to be truncated more by PICLC rather than boosted. Pre-challenge PICLC also could have impacted establishment of the SIV reservoir and this could have uniquely affected SIVwt and SIVΔNef replication. Future studies are needed to address these possibilities. SIVΔNef replication is also a well-recognized determinant of the vaccine effect against pathogenic SIV [70, 78, 96, 97], in agreement with the trend towards better protection alongside higher viral replication in the PICLC-treated SIVΔNef-infected macaques. Mechanistically, SIVΔNef-mediated protection from SIVwt is associated with LN SIV-specific T cell responses and SIVΔNef persistence there [70] as well as non-neutralizing antibody-dependent functions systemically [96] and in the mucosa [98]. Identifying whether and how these correlates were impacted by PICLC was beyond the scope of this study and their study would require future exploration.

Heightened rectal CD169 expression by PICLC did not increase rectal SIV susceptibility *in vivo*. Instead, paralleling expression on CD14^+^ monocytes in blood [82], CD169 was a biomarker of immune activation during pathogenic SIVwt infection in mucosa and LNs. The difference in CD169 levels between SIVwt and SIVΔNef infections agrees with other data on CD169 in animals infected with pathogenic vs. nonpathogenic SIVs [99]. In contrast to CD169, baseline expression of β_7_ correlated with peak viremia. Memory CD4^+^ T cells expressing high levels of α_4_β_7_ are highly susceptible to HIV infection in DC-T cell mixtures *in vitro* [23, 100]; the proportion of these cells correlates with mucosal SIV susceptibility *in vivo* [83]; and antibody blockade of α_4_β_7_ significantly reduces mucosal SIV transmission [101] and therapeutically reduces plasma and gastrointestinal SHIV viral load [102]. Lack of a strong association between baseline β_7_ level and outright infection in this study could simply be due to the fact that we examined β_7_ mRNA in tissue rather than expression of α_4_β_7_ protein on T cells [83]. The correlation between acute viral replication and β_7_ but not CD169 suggests a greater importance of β_7_ in determining mucosal HIV transmission and initial amplification *in vivo*.

Our studies add to a body of work revealing the complex interactions between HIV, DCs, and T cells and how the quality and timing of dsRNA DC maturation dictate downstream events, resulting in a push-pull between blockade to and enhancement of infection. We have shown that PICLC can be used topically at the site of mucosal HIV exposure without promoting infection and potentially reducing it. This opens the door for future applications of PICLC to modulate immunobiology for limiting HIV spread and supports the continued development of PICLC as a vaccine adjuvant.

## Supporting Information

S1 Fig Size differences of dsRNAs utilized in the study. dsRNAs (0.5 μg for all except PICLC which was 10 μg) were separated on an 0.8% agarose gel at 90 V constant. More PICLC was loaded onto the gel since stabilization with poly-L-lysine/carboxymethylcellulose impairs visualization. PIC, PAU, and PICLC were electrophoresed alongside a low molecular weight form of PIC called LMW, as a comparison for a low molecular weight dsRNA species. Band sizes were estimated using the 1kb plus DNA ladder (Invitrogen).
(TIF)

S2 Fig Internalization of potential HIV capture molecules after HIV pulsing. The GMFI of each molecule shown was compared on DCs immediately before and after HIV pulsing (9 donors for CD209 and CCR5; 7 donors for CD206).
(TIF)

S3 Fig Extended DC phenotype. (A) Surface staining and flow cytometry as in Figs 2 and 3 were used to determine the GMFI of the markers shown for iDCs, picDCs, piclcDCs, and pauDCs. (B) Proportion of CCR5^high^ DCs as determined in Fig 3. (C) mRNA RT-qPCR for A3A and A3G performed as in Fig 3.
(TIF)

S4 Fig Antiviral responses in DC-T cell co-cultures. (A) mRNA RT-qPCR was performed for IFN-α, A3A, and A3G as in Fig 3.
(TIF)

S5 Fig *In vitro* activating effect of dsRNAs on human and macaque blood mDCs and CD4^+^ T cells. (A) GMFI is shown for the markers indicated in human blood mDCs. (B) CD69 GMFI in human blood CD4^+^ T cells. (C) CD169 GMFI and percent CD169^high^ cells in macaque blood mDCs parallel findings in human PBMCs.
(TIF)

S6 Fig PICLC acute effects study design. (A) Macaques were administered 1 ml PBS rectally twice 24 hours apart and were then bled and biopsied in the rectal mucosa 4 vs. 24 hours later. After mucosal healing, the macaques were similarly administered 1 mg (in 1ml) PICLC and bled and biopsied. (B) Macaques were biopsied and rested before 2 mg or 4 mg single doses of PICLC were rectally administered. The macaques were biopsied in rectal mucosa 24 hours later.
(TIF)

S7 Fig Rapid bystander activation of blood pDCs in response to rectal PICLC. Blood pDCs in the macaques described in Fig 6 were characterized at the indicated times post-treatment by their frequency (%Lin^-^HLA-DR^+^CD123^+^) and expression of activation markers. *P<0.05, **P<0.01, ***P<0.001.
(TIF)

S8 Fig Rectal PICLC does not induce a systemic pro-inflammatory response. Pro-inflammatory cytokines in the plasma of the macaques described in Fig 6 were measured by Luminex assay (see S1 Methods) in duplicate or triplicate. The post-PICLC (4 hours and 24 hours) data for each animal were normalized against the animal’s post-PBS data and shown as a fold difference vs. baseline. No significant differences were detected.
(TIF)

S9 Fig SIV challenge study schematics. Macaques were treated rectally twice over 24 hours with PICLC (1 mg each dose). They were challenged with SIVmac239 (SIVwt) coincidentally with (coincident, A) or 24 hours after (24h pre, B) the second dose of PICLC and followed for 5 months. (C) Macaques were treated rectally twice over 24 hours with PICLC (1 mg each dose) and challenged with SIVmac239ΔNef (SIVΔNef) 24 hours after the second dose. Twelve weeks later, the macaques were challenged with SIVwt and followed for 5 months.
(TIF)

S10 Fig IFN-α in plasma of PICLC-treated SIVwt challenged macaques. IFN-α levels in plasma of SIVwt, PICLC-treated macaques were measured by ELISA as described in S1 Methods. Black symbols indicate animals challenged coincidentally with PICLC application, and red symbols indicate animals challenged 24 hours after the second PICLC application. Infected and uninfected animals are denoted by closed and open symbols, respectively. The lower limit of quantification of the assay was 25 pg/ml (upper dashed line) and standard curve could be calculated with a low range dilution down to 15 pg/ml (lower dashed line).
(TIF)

S1 Methods Detection of soluble immune factors. (DOCX)

S1 Table Primer sequences for Sybr Green RT-qPCR. (DOCX)
