# Supplementary figures and images for: PolyICLC Exerts Pro- and Anti-HIV Effects on the DC-T Cell Milieu In Vitro and In Vivo
Source: PLoS One. 2016 Sep 7;11(9):e0161730. doi: 10.1371/journal.pone.0161730 (PMC5014349; doi:10.1371/journal.pone.0161730)

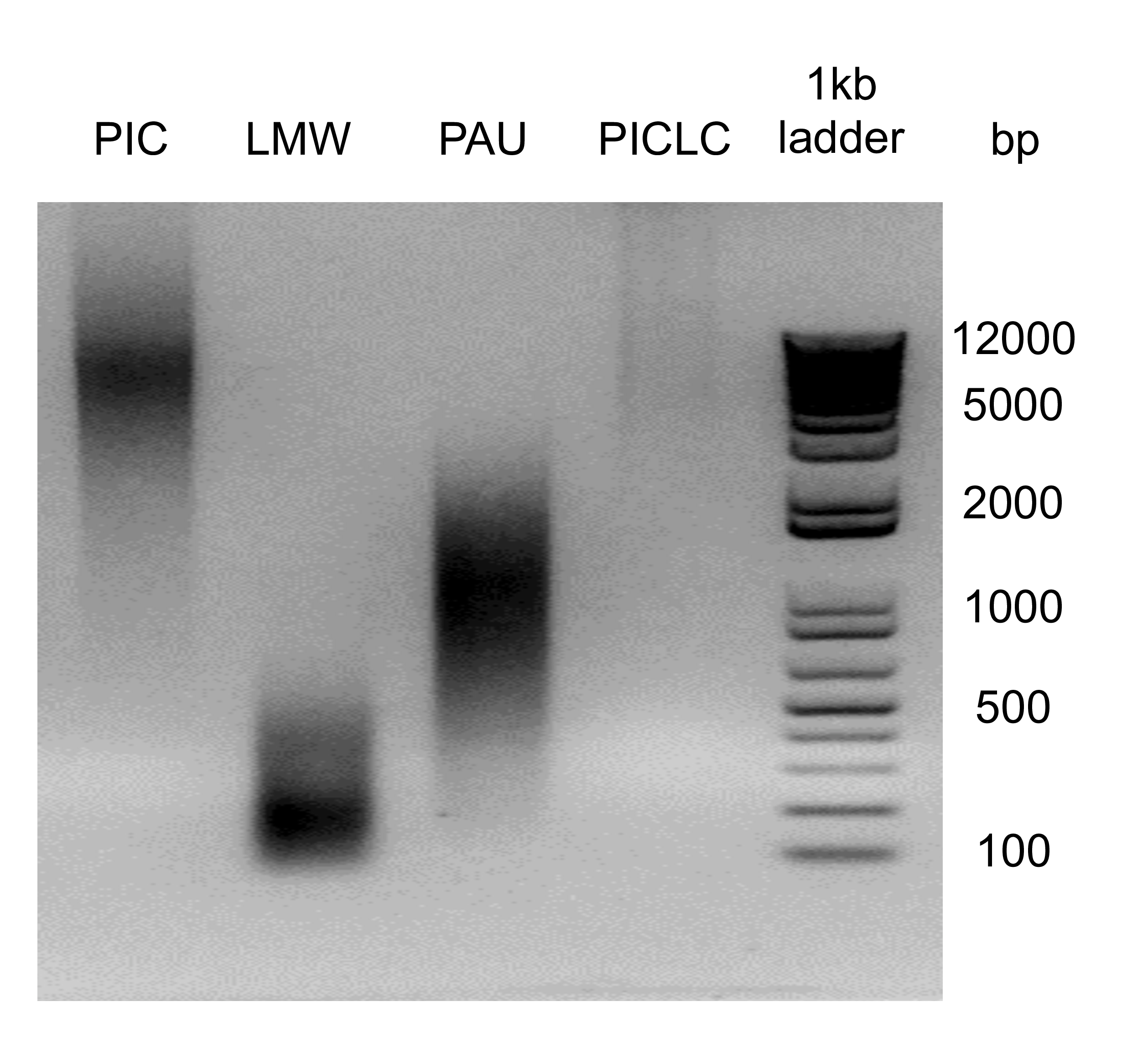

Supplement: S1 Fig — dsRNAs (0.5 μg for all except PICLC which was 10 μg) were separated on an 0.8% agarose gel at 90 V constant. More PICLC was loaded onto the gel since stabilization with poly-L-lysine/carboxymethylcellulose impairs visualization. PIC, PAU, and PICLC were electrophoresed alongside a low molecular weight form of PIC called LMW, as a comparison for a low molecular weight dsRNA species. Band sizes were estimated using the 1kb plus DNA ladder (Invitrogen). (TIF) [file pone.0161730.s001.tif]

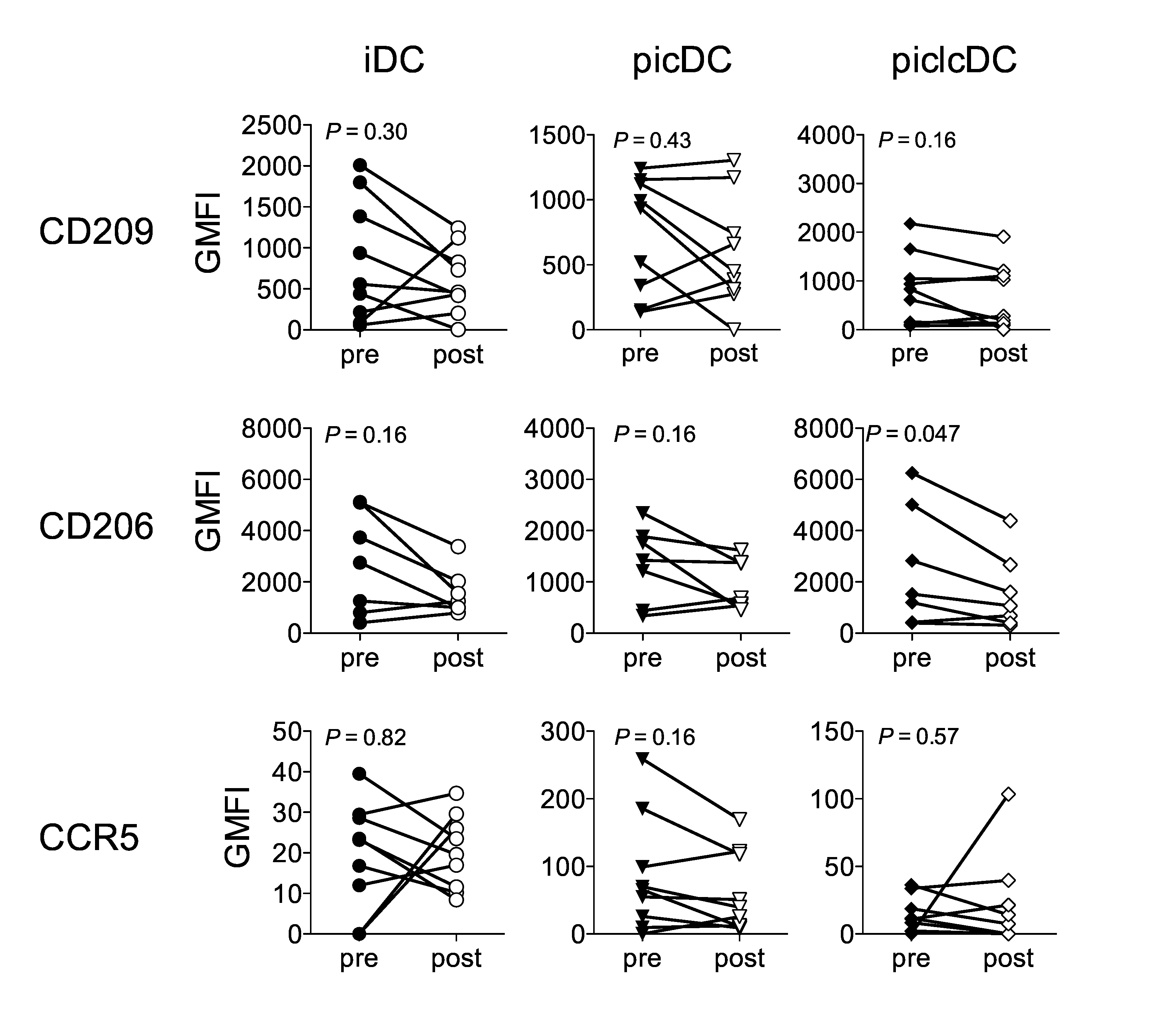

Supplement: S2 Fig — The GMFI of each molecule shown was compared on DCs immediately before and after HIV pulsing (9 donors for CD209 and CCR5; 7 donors for CD206). (TIF) [file pone.0161730.s002.tif]

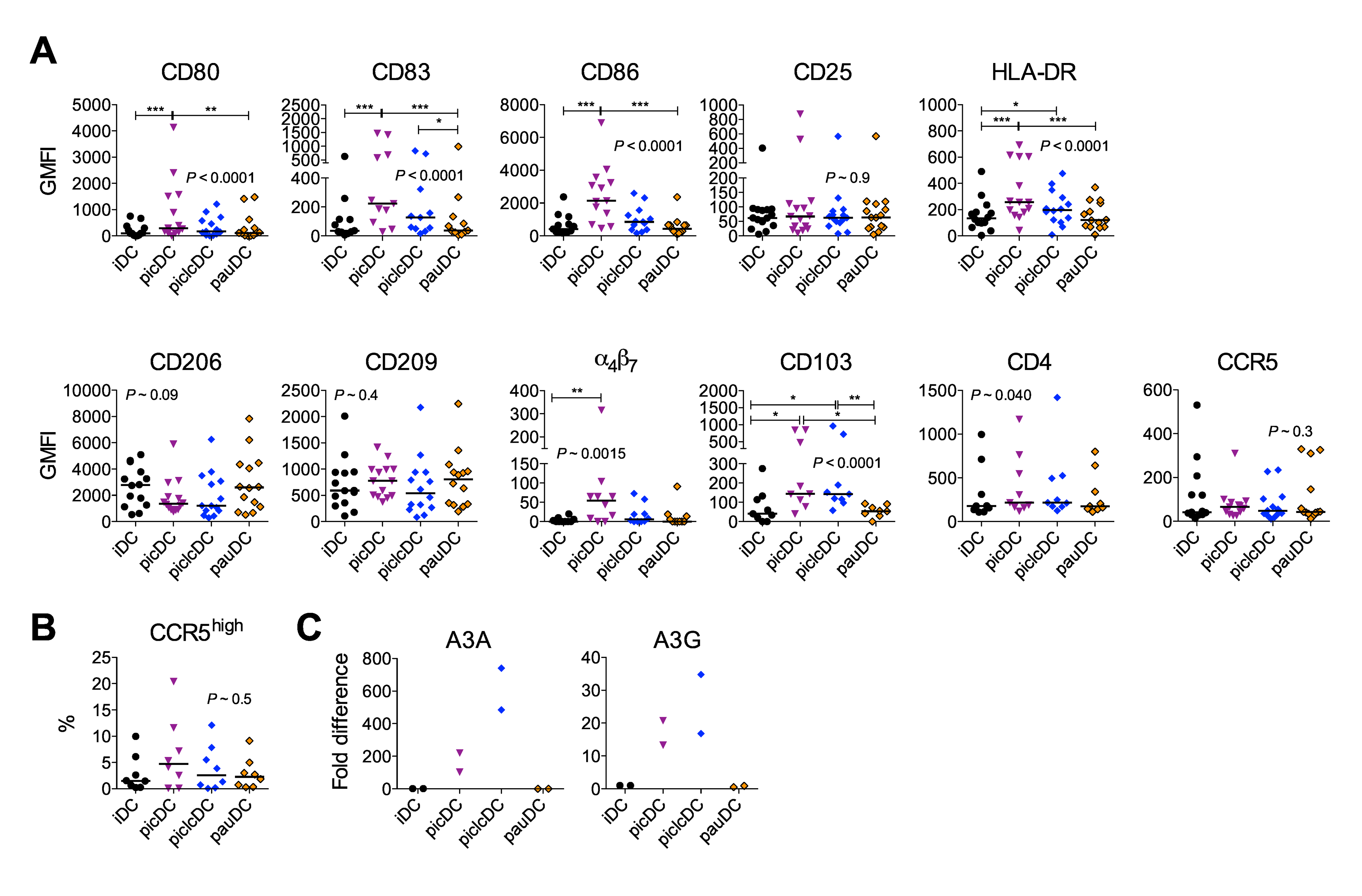

Supplement: S3 Fig — (A) Surface staining and flow cytometry as in Figs 2 and 3 were used to determine the GMFI of the markers shown for iDCs, picDCs, piclcDCs, and pauDCs. (B) Proportion of CCR5high DCs as determined in Fig 3. (C) mRNA RT-qPCR for A3A and A3G performed as in Fig 3. (TIF) [file pone.0161730.s003.tif]

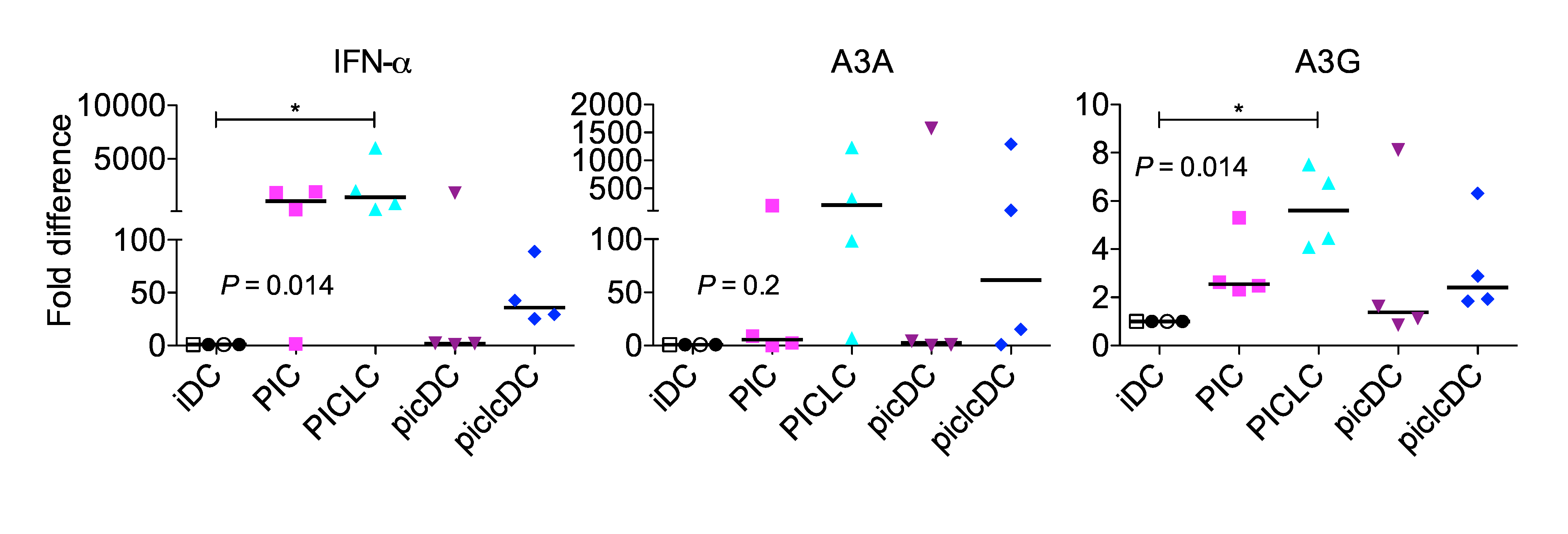

Supplement: S4 Fig — (A) mRNA RT-qPCR was performed for IFN-α, A3A, and A3G as in Fig 3. (TIF) [file pone.0161730.s004.tif]

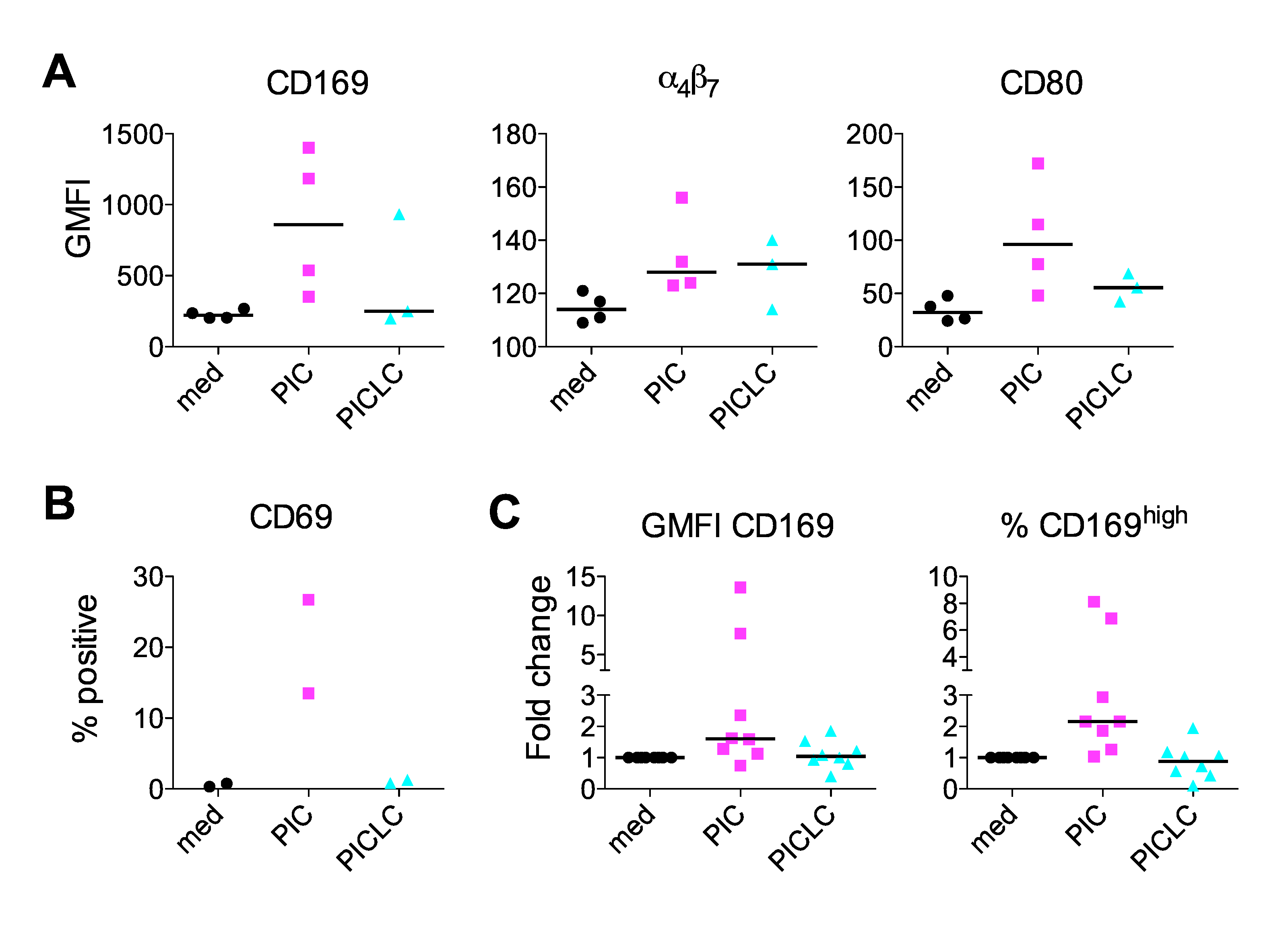

Supplement: S5 Fig — (A) GMFI is shown for the markers indicated in human blood mDCs. (B) CD69 GMFI in human blood CD4+ T cells. (C) CD169 GMFI and percent CD169high cells in macaque blood mDCs parallel findings in human PBMCs. (TIF) [file pone.0161730.s005.tif]

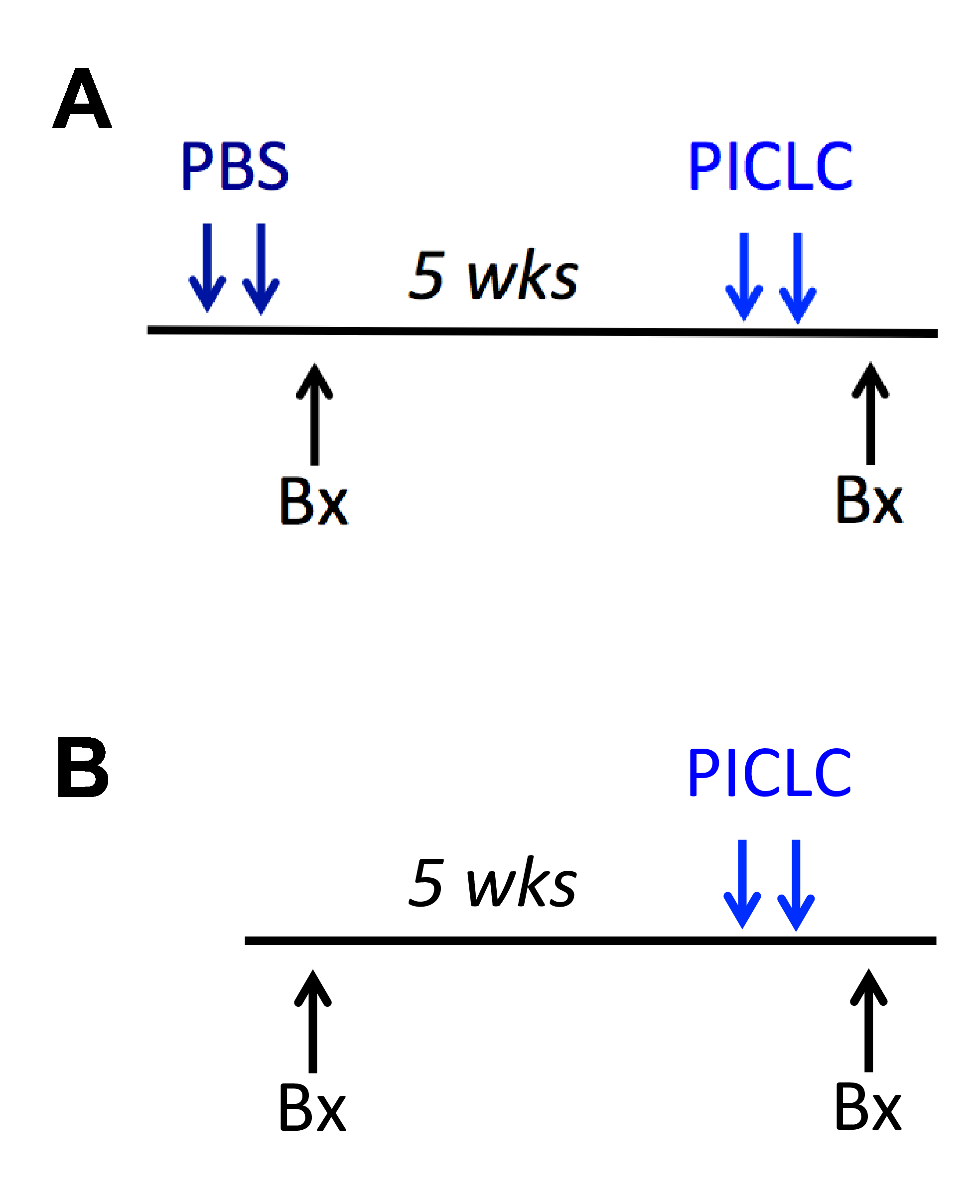

Supplement: S6 Fig — (A) Macaques were administered 1 ml PBS rectally twice 24 hours apart and were then bled and biopsied in the rectal mucosa 4 vs. 24 hours later. After mucosal healing, the macaques were similarly administered 1 mg (in 1ml) PICLC and bled and biopsied. (B) Macaques were biopsied and rested before 2 mg or 4 mg single doses of PICLC were rectally administered. The macaques were biopsied in rectal mucosa 24 hours later. (TIF) [file pone.0161730.s006.tif]

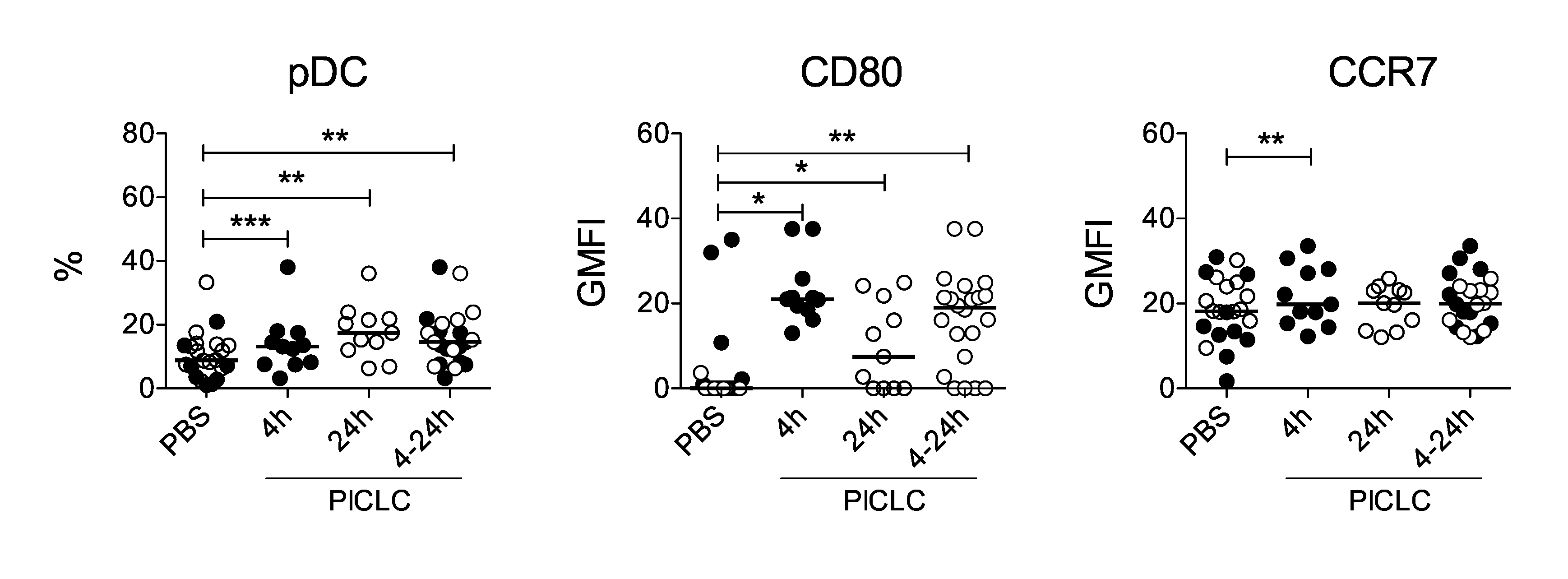

Supplement: S7 Fig — Blood pDCs in the macaques described in Fig 6 were characterized at the indicated times post-treatment by their frequency (%Lin-HLA-DR+CD123+) and expression of activation markers. *P<0.05, **P<0.01, ***P<0.001. (TIF) [file pone.0161730.s007.tif]

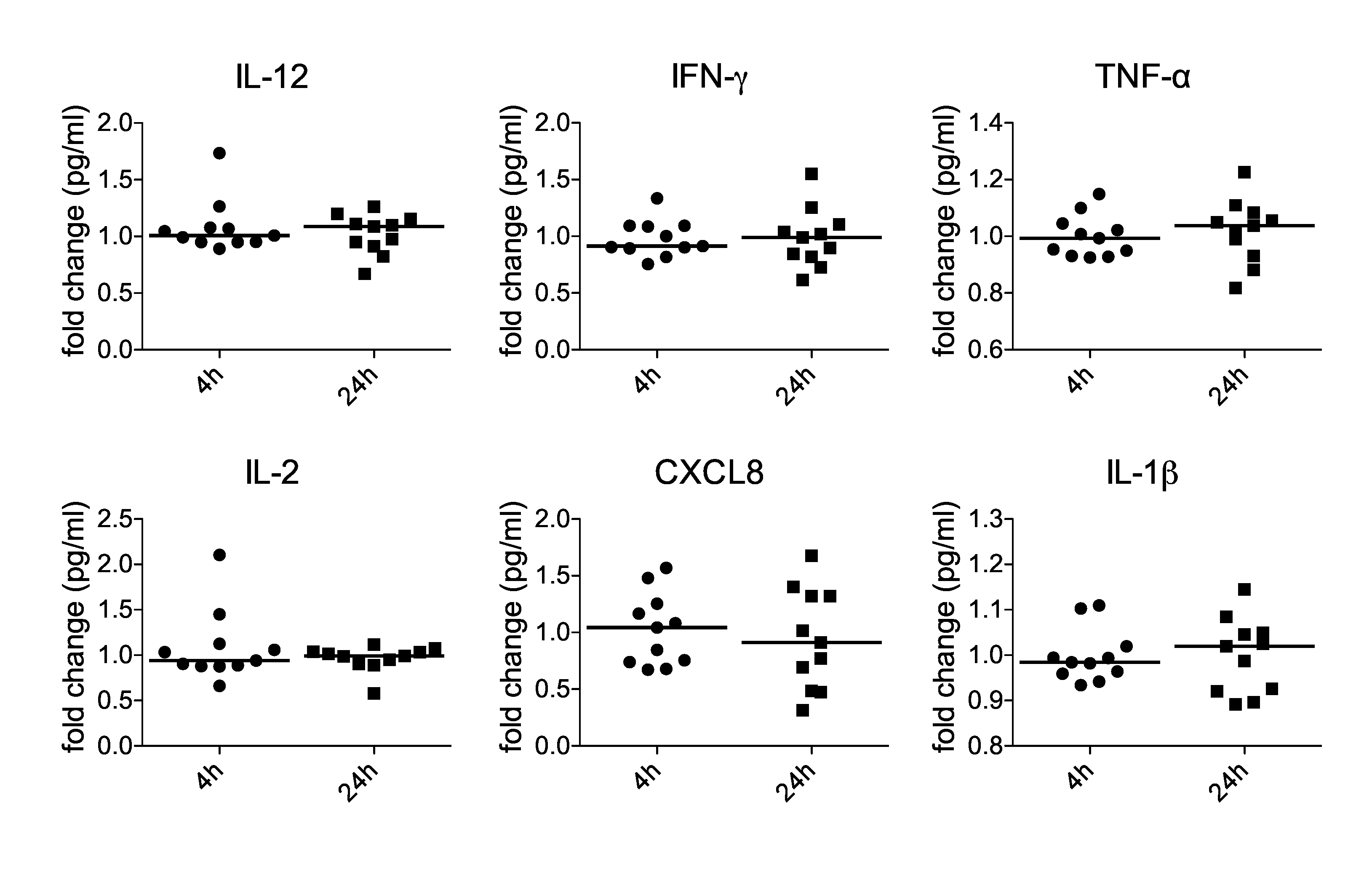

Supplement: S8 Fig — Pro-inflammatory cytokines in the plasma of the macaques described in Fig 6 were measured by Luminex assay (see S1 Methods) in duplicate or triplicate. The post-PICLC (4 hours and 24 hours) data for each animal were normalized against the animal’s post-PBS data and shown as a fold difference vs. baseline. No significant differences were detected. (TIF) [file pone.0161730.s008.tif]

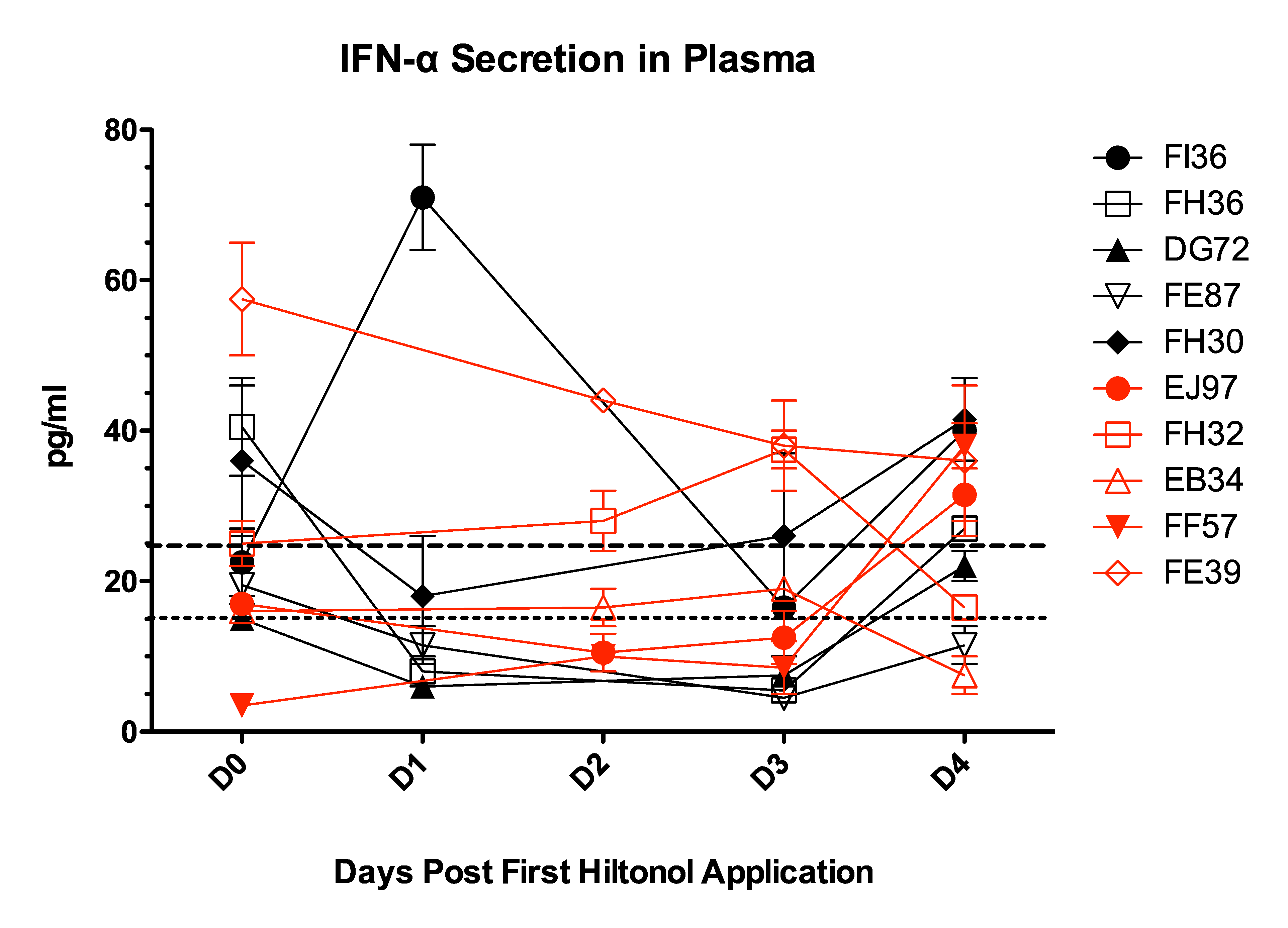

Supplement: S10 Fig — IFN-α levels in plasma of SIVwt, PICLC-treated macaques were measured by ELISA as described in S1 Methods. Black symbols indicate animals challenged coincidentally with PICLC application, and red symbols indicate animals challenged 24 hours after the second PICLC application. Infected and uninfected animals are denoted by closed and open symbols, respectively. The lower limit of quantification of the assay was 25 pg/ml (upper dashed line) and standard curve could be calculated with a low range dilution down to 15 pg/ml (lower dashed line). (TIF) [file pone.0161730.s010.tif]
