# Supplementary material for: PolyICLC Exerts Pro- and Anti-HIV Effects on the DC-T Cell Milieu In Vitro and In Vivo
Source: PLoS One. 2016 Sep 7;11(9):e0161730. doi: 10.1371/journal.pone.0161730 (PMC5014349; doi:10.1371/journal.pone.0161730)
Supplement: S1 Methods — (DOCX) [file pone.0161730.s011.docx]

**S1 Methods**

**Detection of Soluble Immune Factors**

Cytokine and chemokine levels in macaque plasma were measured using the monkey-reactive Beadlyte human 14-plex Detection System (Invitrogen). Data were acquired on a Luminex 200 instrument (Luminex, Austin, TX) and analyzed using StarStation software version 2.0 (Applied Cytometry Systems, Sacramento, CA). IFN-α levels were measured using a commercial ELISA kit (PBL Interferon Source, Piscataway, NJ; lower limit of quantification, 25 pg/ml).
