# Supplementary material for: PolyICLC Exerts Pro- and Anti-HIV Effects on the DC-T Cell Milieu In Vitro and In Vivo
Source: PLoS One. 2016 Sep 7;11(9):e0161730. doi: 10.1371/journal.pone.0161730 (PMC5014349; doi:10.1371/journal.pone.0161730)
Supplement: S1 Table — (DOCX) [file pone.0161730.s012.docx]

**S1 Table. Primer sequences for Sybr Green RT-qPCR**

| Species | Gene | Forward (F)/ Reverse (R) | Sequence |
| --- | --- | --- | --- |
| Human | IFN-α | F | GTCAAGCTGCTCTGTGGGCT |
|  |  | R | CTACCCAGGCTGTGGGTTTG |
|  | A3A | F | TGCCCGCATCTATGATTACG |
|  |  | R | AGCATTTGCAGTGCCTCCTT |
|  | A3G | F | CAATGACACCTGGGTCCTGC |
|  |  | R | TGGTGGCATAGAAAGCCCCT |
|  | CD317 | F | AGCTGGCACATCTTGGAAGG |
|  |  | R | AATGTTCAAGCGAAAAGCCG |
|  | CD169 | F | AGCGATGCTGGCGTCTACA |
|  |  | R | TGAGACCAAAGAGCCCACG |
|  | RPL-19 | F | TTTGGGTATGCTCAGGCTTCA |
|  |  | R | CAGCGGAGGACACTAGAGGC |
| Rhesus macaque | IFN-α | F | TTGCTTTACTGGTGGCCCTG |
|  |  | R | GAGAGCAGCTTGACTTGCAGC |
|  | IFN-β | F | CCTGTTGTGCTTCTCCACTACG |
|  |  | R | CCAAGCAAGTTGTAGCTCATGG |
|  | A3A | F | TTCCCTGTGGTGATTACGGC |
|  |  | R | CCTCACACAGGAAGCGCA |
|  | A3G | F | TTGGGTCAGTGGACAGCATG |
|  |  | R | AGGCGCTCCACCTTGTAACA |
|  | CD169 | F | TACAAGAACCATGTCCCGCTG |
|  |  | R | GCCCTAGTGACGGAATGCAG |
|  | β7 | F | GCAACCATGTCCAAATCAACC |
|  |  | R | GCTTGGAGGGAAACCCAGA |
|  | MAdCAM-1 | F | CTTGTGTACGCCTTCCCGGACCAGC |
|  |  | R | ACAGGCCACCTCCGGGTCAC |
|  | GAPDH | F | GCCGCATTTTCTCTTGCATC |
|  |  | R | CTTCCCCATGGTGTCTCAGG |
